# An Alternative Self-Splicing Intron Lifecycle Revealed by Dynamic Intron Turnover in *Epichloë* Endophyte Mitochondrial Genomes

**DOI:** 10.1093/molbev/msaf076

**Published:** 2025-04-02

**Authors:** Jennie Chan, Mauro Truglio, Christopher L Schardl, Murray P Cox, Carolyn A Young, Austen R D Ganley

**Affiliations:** School of Biological Sciences, University of Auckland, Auckland, New Zealand; Microbiology and Virology Unit, San Gallicano Dermatological Institute, IRCCS, Istituti Fisioterapici Ospitalieri (IFO), Rome, Italy; Department of Plant Pathology, University of Kentucky, Lexington, KY 40546, USA; School of Natural Sciences, Massey University, Palmerston North, New Zealand; Noble Research Institute, Ardmore, OK 73401, USA; Entomology and Plant Pathology, North Carolina State University, Raleigh, NC 27695, USA; School of Biological Sciences, University of Auckland, Auckland, New Zealand; Digital Life Institute, University of Auckland, Auckland, New Zealand

**Keywords:** intron, self-splicing, epichloe, mitochondrial genome, homing endonuclease

## Abstract

Self-splicing group I and II introns are selfish genetic elements that are widely yet patchily distributed across the tree of life. Their selfish behavior comes from super-Mendelian inheritance behaviors, collectively called “homing”, which allow them to rapidly spread within populations to the specific genomic sites they home into. Observations of self-splicing intron evolutionary dynamics have led to the formulation of an intron “lifecycle” model where, once fixed in a population, the introns lose selection for homing and undergo an extensive period of degradation until their eventual loss. Here, we find that self-splicing introns are common in the mitochondrial genomes of *Epichloë* species, endophytic fungi that live in symbioses with grasses. However, these introns show substantial intron presence–absence polymorphism, with our analyses suggesting that these result from a combination of vertical intron inheritance coupled with multiple invasion and loss events over the course of *Epichloë* evolution. Surprisingly, we find little evidence for the extensive intron degradation expected under the existing intron lifecycle model. Instead, these introns in *Epichloë* appear to be lost soon after fixation, suggesting that *Epichloë* self-splicing introns have a different lifecycle. However, rapid intron loss alone cannot explain our results, indicating that additional factors, such as the evolution of homing suppressors, also contribute to *Epichloë* self-splicing intron dynamics. This work shows that self-splicing introns have more diverse evolutionary dynamics than previously appreciated.

## Introduction

Introns are segments of DNA that interrupt coding regions of genes and are spliced out from RNA to reconstitute the coded element. Self-splicing introns are a major class of intron that typically encode the ability to splice themselves out of their RNA host (Lambowitz and Belfort 1993; Hausner 2012), unlike the better-known spliceosomal introns that are entirely dependent on exogenous factors for splicing. Self-splicing introns are sporadically distributed across bacteria, archaea, and eukaryotes (predominately in mitochondrial and chloroplast genomes; Haugen et al. 2005; Zimmerly and Semper 2015; Nawrocki et al. 2018), making them phylogenetically more widespread than the eukaryote-specific spliceosomal introns. There are two major self-splicing intron types, group I and group II introns (Lambowitz and Belfort 1993). These have highly diverse primary sequences and sizes, but each intron type folds into a conserved secondary structure that facilitates the self-splicing reaction. Group I and II introns differ in both their secondary structure and splicing mechanism, with group II introns typically requiring protein factors to assist with splicing in vivo (Lambowitz and Zimmerly 2004; Lang et al. 2007).

**Fig. 1.**
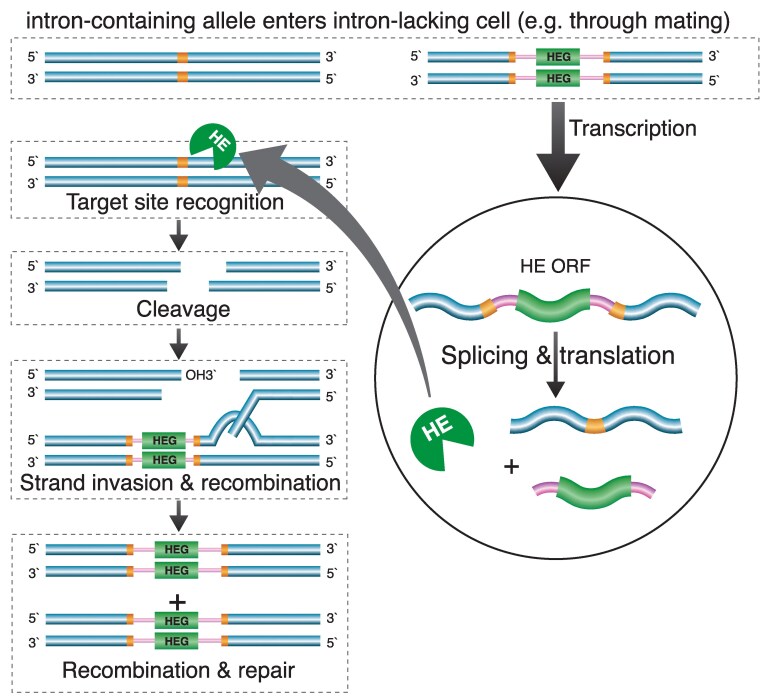
Homing reaction of group I self-splicing introns. Presence of intron-containing and intron-less copies of the host gene (blue) in a cell (e.g. following mating) leads to intron homing. The intron (pink) self-splices from the host transcript (wavy line) following transcription, and the intron's homing endonuclease (green) gene (HEG) is translated to produce a homing endonuclease (HE). The HE cleaves its recognition sequence (orange) in the intron-less gene, initiating double-strand break recombination (only shown for one of the two strands). Recombination uses the intron-containing gene as the template to repair the break in the intron-less copy, thus copying the intron into the intron-less gene.

Group I and II introns are considered to be selfish genetic elements because they mediate their own spread through host populations whilst typically not providing any benefit to the host (Dujon 1989). This ability to spread depends on “homing”, which refers to the lateral transfer of an intron from one host gene to a homologous copy of the gene lacking the intron (Dujon 1989). Homing is exemplified by group I introns, which encode a homing endonuclease. Homing endonucleases induce a double-strand break (DSB) at a specific DNA sequence in the host gene, typically 15 to 20 bp long, which constitutes the site of intron insertion (Burt and Koufopanou 2004; Hedberg and Johansen 2013). Events such as mating that combine an intron-containing gene with an intron-less gene result in cleavage of the intron-less gene by the homing endonuclease whereas the intron-containing gene is protected from cleavage by the intron's presence. The intron-containing gene then serves as the template for DSB repair by homologous recombination, resulting in the intron being copied into the intron-less gene (Fig. 1), often with co-conversion of a few nucleotides of the sequence flanking the intron (Dujon 1989). Group II introns employ a different homing mechanism mediated by proteins unrelated to group I homing endonucleases that instead facilitate splicing and mobility through a “retro-homing” process that involves insertion of the intron RNA into an intron-less gene through reverse transcriptase activity (Zimmerly and Semper 2015). The homing activity conferred on an intron by a homing gene can result in “super-Mendelian” spread of the intron through a population (Koufopanou et al. 2002).

Once a self-splicing intron reaches fixation in a population, homing gene activity is no longer under selection and can be lost. Loss of homing activity due to mutation has been observed to then be followed by a long degradation phase that ends with entire intron loss (e.g. Goddard and Burt 1999; Haugen et al. 2004; Ferandon et al. 2010; Thiery et al. 2010; Mardanov et al. 2014; Wu and Hao 2014). Although not experimentally confirmed, the most likely mechanism of entire intron loss is retroprocessing, where reverse transcription of an mRNA transcript from which the intron has been spliced out of is followed by homologous integration into the genome to replace the intron-containing gene (Dujon 1989). This has led to the formulation of self-splicing intron “lifecycle” (Goddard and Burt 1999), where rapid homing of an intron into a population lacking the intron and its subsequent fixation are followed by a slow period of intron degradation. The cycle is completed once the intron is completely lost, as this leaves the intron site vacant for a subsequent round of invasion (Goddard and Burt 1999).

*Epichloë* is a genus of ascomycete fungi that form endophytic associations with Pooideae (‘cool-season” grasses). These associations are mutualistic, involving production of secondary metabolites that help protect the host, for example, from grazing herbivores (Schardl et al. 2009; Caradus and Johnson 2020). In *Epichloë*, both sexual (teleomorph) and asexual (anamorph) forms have been documented, and interspecific hybridization often results in strictly asexual allopolyploid (hybrid) species (Moon et al. 2004). Asexual *Epichloë* grow asymptomatically in the intercellular spaces of stems, leaves, inflorescences, and seeds of their host, and they propagate clonally through host seeds. Sexual *Epichloë* species also grow asymptomatically in their hosts but can act as “replacement pathogens” because their sexual cycle suppresses maturation of host inflorescences, a symptom known as choke disease (Alderman et al. 1997). Many *Epichloë* species can reproduce both asexually and sexually, thus the effects of *Epichloë*-grass symbioses range from mutualism to antagonism (Schardl et al. 2004). The ecological and economic importance of *Epichloë* species that results from the symbiotic relationship with pasture grasses has made them a model system for studying plant–microbe interactions (Saikkonen et al. 2016).

The mitochondrial DNA (mtDNA) genomes of some *Epichloë* species have been reported to harbor group I introns (Campbell et al. 2017), a feature common in mtDNA genomes of fungi. Fungal group I introns typically harbor GIY-YIG and LAGLIDAG family homing endonucleases (Megarioti and Kouvelis 2020). Group II introns have also been reported in mtDNA genomes of some fungi (Hausner 2003). Self-splicing fungal mtDNA introns often display high levels of presence/absence polymorphism (Sandor et al. 2018), even within species (e.g. de Zamaroczy and Bernardi 1985; Belcour et al. 1997; Jung et al. 2012; Freel et al. 2014; Wang et al. 2018; Fan et al. 2019; Ponts et al. 2020; Mayers et al. 2021; Zubaer et al. 2021; Powell et al. 2023). We became interested in the evolutionary dynamics of mtDNA self-splicing introns in *Epichloë* when, during whole genome sequencing of members of the genus, we found that intron presence/absence polymorphism made aligning mtDNA contigs between isolates difficult. Here, we characterized the self-splicing intron profile of mtDNA genomes across the genus *Epichloë* to determine the nature of this intron presence/absence polymorphism. We found many introns, including both group I and group II introns, colonizing multiple insertion sites in multiple mtDNA protein-coding genes, but with high levels of intron presence/absence polymorphism. We infer the occurrence of intron invasion and loss but find little evidence for the long intron degradation phase predicted by the canonical intron lifecycle. These results suggest that intron dynamics in *Epichloë* mtDNA are characterized by very rapid purging of self-splicing introns following their fixation.

## Results

### Extensive Self-splicing Intron Presence-absence Polymorphism Across the *Epichloë* Genus

To comprehensively characterize *Epichloë* mtDNA self-splicing introns, we obtained full-length mtDNA genomes from 33 *Epichloë* isolates representing 13 species across the genus (supplementary table S1, Supplementary Material online). We used MFannot to annotate the mitocohondrial protein-coding genes and their introns. All mtDNA genomes contained 14 protein-coding genes (outside of the ribosomal RNA genes), as previously reported for Hypocrealean fungi (Pantou et al. 2008; Campbell et al. 2017), and gene order is the same in all cases (supplementary fig. S1, Supplementary Material online). We found a total of 641 self-splicing introns occupying 38 different insertion sites in eleven of the protein-coding genes across all *Epichloë* mtDNA genomes (supplementary table S2, Supplementary Material online). Of these, 8 sites in 4 genes are occupied by group II introns and 30 sites in 11 genes by group I introns, with MFannot further dividing the group I introns into 5 different sub-classes (IB, IC1, IC2, ID, and I[d; derived]). The introns range in size from 910 to 2,575 bp (supplementary Data S1, Supplementary Material online). Comparison of intron site occupancy across isolates revealed substantial polymorphism: each isolate contains between 10 and 30 introns, and introns are present in anywhere between one isolate (two introns: cob_201 and cox3_203) and all 33 isolates (two introns: nad1_636 and nad5_570; supplementary table S2, Supplementary Material online).

**Fig. 2.**
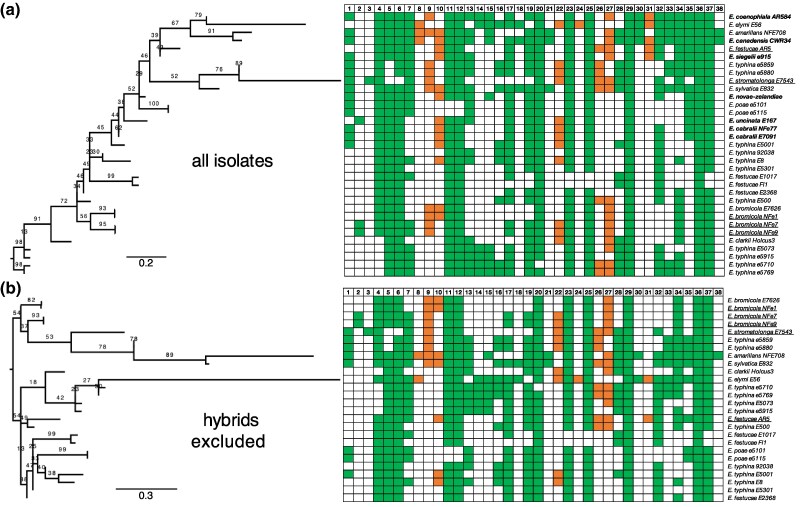
Self-splicing intron presence/absence is patchily distributed amongst *Epichloë* isolates. On the left are dendrograms of *Epichloë* isolates made using intron presence/absence as the character with all isolates included a) and hybrids excluded b). To the right are the corresponding intron presence/absence matrices. Filled spaces indicate intron presence, empty spaces intron absence (green are group I introns; orange are group II introns). Introns are numbered at top according to supplementary table S2, Supplementary Material online. Isolate names are shown at far right, with hybrids indicated in bold and non-hybrid putative asexual species underlined. Support values are bootstraps, and distances are indicated below each dendrogram.

To investigate the evolutionary dynamics of these self-splicing introns, we constructed nuclear gene phylogenies of the *Epichloë* isolates (supplementary Data S2, Supplementary Material online) and mapped intron presence/absence onto these. The distribution of intron presence/absence on these phylogenies is patchy (supplementary fig. S2, Supplementary Material online) and not consistent with a single introduction followed by vertical inheritance for every intron. To test whether this patchiness is the consequence of the mitochondrial genome having a different evolutionary history to the nuclear genome, we constructed a phylogeny from concatenated mtDNA protein-coding genes. However, while the tree topology is different, intron presence/absence is still patchily distributed (supplementary fig. S3, Supplementary Material online). We wondered whether the patchiness is due to hybrid isolates (Leuchtmann et al. 2019; Schardl et al. 2025), but removing the impact of hybrid isolates still resulted in patchy distributions of introns (supplementary fig. S2 and S3, Supplementary Material online). To test whether any phylogenetic topology is consistent with simple vertical inheritance of these introns, we used intron presence/absence as the character to construct dendrograms. However, even this resulted in a patchy intron distribution regardless of whether hybrid isolates were included or excluded (Fig. 2), indicating no phylogeny is consistent with a simple model of intron introduction and vertical inheritance. To quantify the inconsistency between intron distribution and the presence/absence dendrogram, we used ancestral state reconstruction (ASR) to measure consistency indices (CIs), which are the number of observed state changes relative to the minimum possible number (Kluge and Farris 1969). We found that overall CI is low (0.339, vs. 1 for perfect concordance; supplementary table S3, Supplementary Material online). Therefore, we conclude that most of these *Epichloë* self-splicing introns do not follow a simple evolutionary history of a single introduction followed by vertical inheritance.

### Patchy Self-splicing Intron Distribution is Not Explained by Mode of *Epichloë* Reproduction

**Fig. 3.**
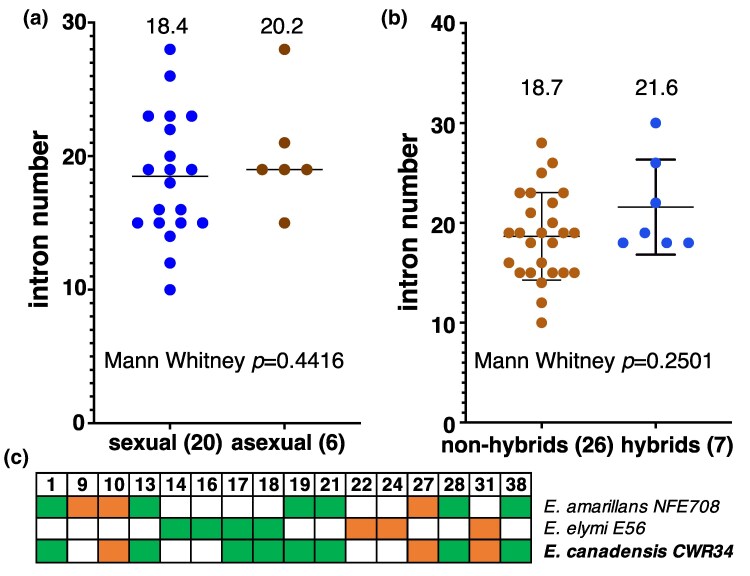
Intron number is not associated with *Epichloë* sexual status or hybridity. Intron numbers for a) non-hybrid sexual and putatively asexual isolates, and b) hybrids and non-hybrids are plotted. Mean values are shown above, *P*-values below, and the number of isolates in each class is indicated in parentheses. Hybrid, non-hybrid, and putatively asexual isolates are indicated in Fig. 2. c) Introns are not always inherited in hybrids. Intron presence/absence from Fig. 2 is indicated for the hybrid *E. canadensis* and its two extant parental species. Introns present or absent in all 3 species are not depicted. See also supplementary fig. S4, Supplementary Material online.

Exclusion of hybrid isolates from the phylogenies did not resolve intron patchiness; however, we wondered whether the reproductive history diversity of *Epichloë*, which includes sexual and asexual species as well as hybrids, contributes to the observed patchiness. One expectation is for introns to be more prevalent in sexual than asexual isolates, because intron spread via homing is thought to be facilitated by sexual mating (Burt and Koufopanou 2004). To test this, we first compared the total number of introns present in sexual versus asexual isolates. Non-hybrid isolates were classed as asexual following (Berry et al. 2022), as were *E. bromicola* isolates NFe7 and NFe9, which are phylogenetically very closely related to *E. bromicola* NFe1 (Yi et al. 2018). Surprisingly, these putatively asexual isolates do not have fewer introns on average than sexual isolates (Fig. 3a), a result that is robust to *E. bromicola* isolates NFe7 and NFe9 being classed as sexual (supplementary table S4, Supplementary Material online).

Asexual designations, other than for hybrids, are tentative as it is difficult to be sure an isolate is asexual (Berry et al. 2022), and we do not know when an isolate became asexual. Therefore, we next compared intron number between hybrid and non-hybrid isolates, given that the fusions that create hybrids should result in them having more introns than non-hybrid isolates as a consequence of homing. However, while intron number is slightly higher in hybrids, the difference is not significant (Fig. 3b). To investigate this further, we directly examined parent–hybrid intron inheritance. This was only possible for one hybrid (*E. canadensis*) because we lack data for one or more parental species for the other hybrids. We found the *E. canadensis* mitochondrial genome is a mosaic of SNPs from its two parental genomes (*E. amarillans* and *E. elymi*; supplementary fig. S4, Supplementary Material online; supplementary Supplementary Data S2, Supplementary material online). The mosaic nature of the hybrid genome may result from recombination that was triggered by homing endonucleases from the 16 introns that are only present in one parent cutting the other parent's mitochondrial genome during a period of heteroplasmy following the hybridization event, although cuts predicted from intron polymorphism can only explain a minority of inferred recombination events (supplementary fig. S4, Supplementary Material online). Moreover, only 11 of the 16 introns present in just one parental genome are found in the mosaic hybrid genome (Fig. 3c), meaning that almost one-third of polymorphic introns (5/16) were not inherited in the hybrid even though its mitochondrial genome would have been susceptible to homing by these introns (Fig. 3c). While it is possible the true parental isolates had different intron compositions to the *E. amarillans* and *E. elymi* isolates analyzed here, all 19 introns present in both parents are present in the hybrid and no introns absent from both parents are present in the hybrid (see supplementary table S2, Supplementary Material online). Therefore, our results suggest that intron homing during hybridization is surprisingly inefficient, which may explain why no significant difference in intron content between hybrids and non-hybrids was observed. Altogether, our results provide no evidence that the patchy intron distribution seen across *Epichloë* isolates is strongly dependent on the mode of reproduction.

### Introns are Predominantly Vertically Inherited With Some Potential Intron Invasions

A possible explanation for the introns not following a simple pattern of a single introduction followed by vertical inheritance is some have jumped to different sites in the mitochondrial genome during *Epichloë* evolution (Hoshina and Imamura 2009). Examination of the 5 bp either side of the group I intron insertion sites showed that all sites have a unique sequence and thus are not likely to have been homing targets for introns from other sites. Furthermore, a phylogeny made from an alignment of the introns in this study showed that introns from each insertion site all form their own monophyletic clade (supplementary fig. S5, Supplementary Material online). Thus, dynamic turnover of introns during the course of *Epichloë* evolution appears to have occurred without existing introns colonizing new sites in the mtDNA genome, similar to what has been concluded previously (e.g. Bhattacharya et al. 2002; Fan et al. 2019).

Our results to date suggest that most self-splicing *Epichloë* introns do not follow a simple vertical inheritance pattern, implying that intron invasions and/or losses have occurred during *Epichloë* evolution. To investigate this, we used the ASRs to quantify the likely extent of intron invasion and/or loss. The ASRs inferred ∼1.5 gains and 0.88 to 1.29 losses per intron, depending whether hybrids are included (Table 1). We also determined the numbers when only invasions or only losses are allowed from the ASRs to examine the likelihood of extreme scenarios of only invasions (i.e. frequent independent invasions) or only losses (i.e. all introns were present in the last *Epichloë* common ancestor). We found that many more events are required than when both invasions and losses are allowed (Table 1). Similar results were obtained with a different ASR tool, RASP (supplementary Data S2, Supplementary Material online), suggesting the results are robust. Therefore, only-invasion and only-loss scenarios are possible but require substantially more events than if both have occurred with equal probability. Together, the ASRs infer that multiple invasions and losses most parsimoniously explain observed intron distribution.

**Table 1:** Number of intron gain/loss events inferred from Mesquite ancestral state reconstructions

Intron	ASR from intron presence/absence dendrograms							
	Gains		Losses		Only gains^a^		Only losses	
	Hybrids	No hybrids	Hybrids	No hybrids	Hybrids	No hybrids	Hybrids	No hybrids
**1. atp6_521**	1	3	3	0	6	3	9	8
**2. atp6_578**	2	1	0	0	2	1	10	3
**3. cob_201**	1	1	0	0	1	1	13	4
**4. cob_393**	1	1	0	1	1	3	3	3
**5. cob_490**	0	0/1 (0.5)^b^	1	1/2 (1.5)	12	4	1	2
**6. cob_506**	0	0	1	1	13	4	1	1
**7. cob_823**	4	6	1	0	6	6	10	8
**8. cox1_50**	1/2 (1.5)	2	0/1 (0.5)	0	2	2	13	9
**9. cox1_108**	4	0/1 (0.5)	0	0/1 (0.5)	4	1	13	1
**10. cox1_199**	2/3 (2.5)	4	3/4 (3.5)	0	9	4	11	9
**11. cox1_212**	0	0	1	1	11	5	1	1
**12. cox1_281**	0	0	1	1	11	5	1	1
**13. cox1_615**	2	3	4	2	11	7	8	8
**14. cox1_709**	1	1	1	0	3	1	11	3
**15. cox1_731**	2	2	1	0	4	2	10	7
**16. cox1_867**	4/7 (5.5)	4/5 (4.5)	0/3 (1.5)	0/1 (0.5)	7	5	12	10
**17. cox1_1057**	0	0	5	5	16	9	5	5
**18. cox1_1125**	3	2	0	0	3	2	14	9
**19. cox1_1262**	0	0	4	4	16	9	4	4
**20. cox2_228**	0	0	1	1	2	5	1	1
**21. cox2_357**	2	1	0	0	2	1	13	5
**22. cox2_373**	5/6 (5.5)	4	1/2 (1.5)	1	8	6	12	9
**23. cox2_651**	0	0	1	1	6	5	1	1
**24. cox3_203**	1	1	0	0	1	1	14	5
**25. cox3_216**	0	0	1	1	6	5	1	1
**26. cox3_276**	3/4 (3.5)	3	0/1 (0.5)	1	4	5	10	7
**27. cox3_471**	1	0	4	4	10	8	9	4
**28. nad1_144**	4	4	1	1	7	6	8	9
**29. nad1_636**	0	0	0	0	1	1	0	0
**30. nad2_378**	1	2	0	0	1	2	12	9
**31. nad2_570**	1	2	1	0	3	2	12	6
**32. nad2_1647**	0/2 (1)	0/4 (2.14)	3/5 (4)	1/5 (2.85)	11	7	5	5
**33. nad4_505**	1/4 (2.5)	2	0/3 (1.5)	0	4	2	11	6
**34. nad4L_239**	2	1	2	2	7	7	7	5
**35. nad5_426**	1	4	1	0	6	4	8	10
**36. nad5_570**	0	0	0	0	1	1	0	0
**37. nad5_717**	0	0	1	1	6	5	1	1
**38. nad6_233**	1	1	0	0	1	1	13	6
**Total**	**57**	**58.64**	**49**	**33.35**	**225**	**148**	**288**	**186**
**Total per intron**	**1.50**	**1.54**	**1.29**	**0.88**	**5.92**	**3.89**	**7.58**	**4.89**

^a^Values are the numbers of gains (or losses in adjacent columns) when only gains (or losses) are allowed.

^b^Multiple values represent uncertainties and are presented as min/max (mean) number of events. In case of complex uncertainties, several possible mappings are considered and the value is the mean of all these (rather than just the mean of the min/max).

**Fig. 4.**
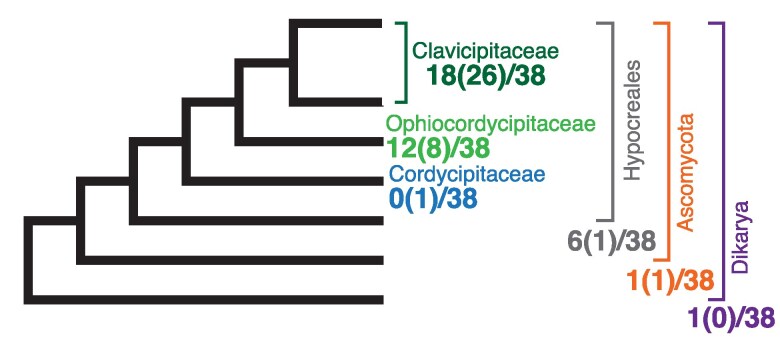
Most self-splicing introns in *Epichloë* are similar to introns from other closely-related fungi. How many of the 38 introns the closest blastn match fall into each taxonomic group is indicated on the schematic fungal phylogeny. Numbers in parentheses are for the closest taxonomic group to *Epichloë* when the top 10 blastn matches are considered. See supplementary table S5, Supplementary Material online for more details. Phylogeny is not to scale.

We next looked to see whether our data could provide additional evidence for the scale of intron invasion and/or loss. First, we looked at the taxonomic origin of each intron, as if there have been multiple invasions the sources might differ. We performed blastn searches using the consensus sequences from alignments of each intron (supplementary Data S1, Supplementary Material online) as queries. The closest match for every intron was from a fungal mitochondrial genome, with 26 of the 38 introns having one of their 10 closest matches with a member of the same family as *Epichloë* (the Clavicipitaceae) and all but one (cox2_373) being from the same order (the Hypocreales; Fig. 4; supplementary table S5, Supplementary Material online). Therefore, all introns are most closely related to other fungal mitochondrial introns, albeit from different fungal species.

To identify potential instances of horizontal transfer, we looked at introns with low nucleotide sequence identities. We first identified nine introns where some isolates had particularly high nucleotide divergences (supplementary table_S5, Supplementary Material online). To examine whether these might result from independent intron invasions, we used blastn to see if the closest match differed for divergent intron types when present, but only found this for two of the nine introns (cob_490 and cox1_1125). We also constructed phylogenetic trees of these 9 introns plus 3 others where nucleotide sequence identity between isolates went below 96% (supplementary table S5, Supplementary Material online), as nesting of a non-*Epichloë* intron within *Epichloë* might indicate an invasion. We found strong supported for nesting of the closest-matching intron for cox1_1125, weak support for cob_490, cox1_108, cox3_216, nad2_1647, nad4_505 and nad5_717, and no support for the remaining introns (supplementary Information S1, Supplementary Material online). Thus, there is some support for a small number of independent intron invasions into *Epichloë*. The average number of losses from these introns is less than the overall average (Table 1), though, suggesting these introns do not disproportionately contribute to the ASR inference of intron loss.

**Fig. 5.**
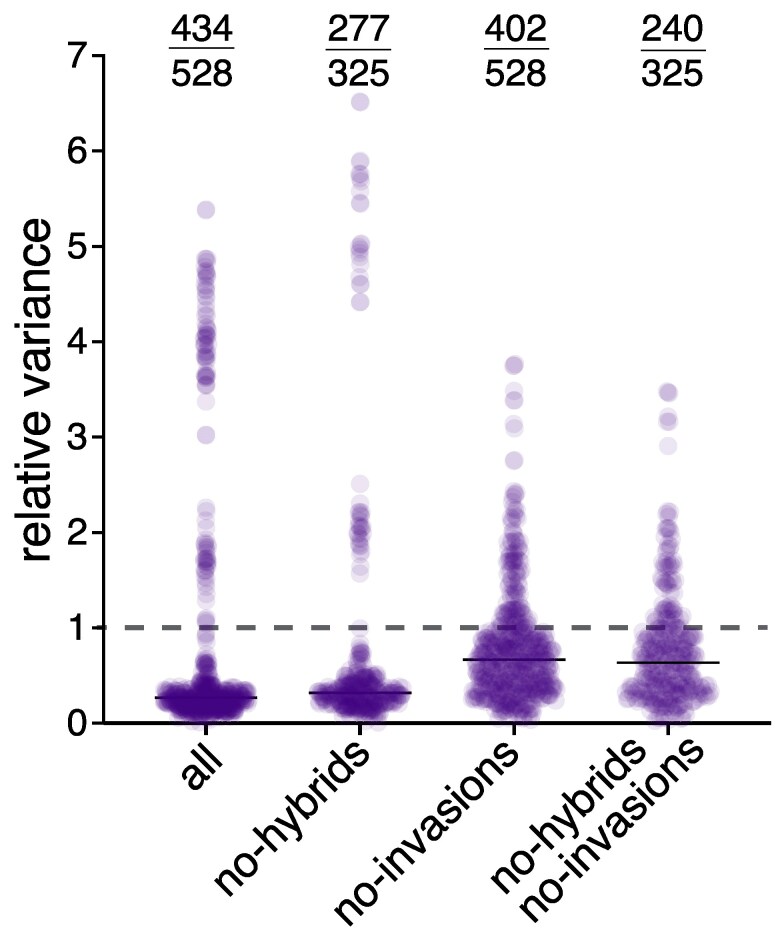
Intron divergences are consistent with vertical transmission with some invasion and loss. Relative variance in intron nucleotide identity across introns is plotted, with each point being an isolate pair. Nucleotide identities for each intron were normalized to the mean % identity for that intron across all isolate pairs and variance is relative to intron nucleotide divergence variance across the whole dataset, which is thus 1 (dotted line). Shown are variances for all isolate pairs with and without hybrids, and with and without the 8 introns showing evidence of invasion (cob_490, cob_506, cox1_108, cox1_1125, cox3_216, nad2_1647, nad4_505 and nad5_717), as indicated. Black horizontal bars are mean relative variances for each dataset; values at top are numbers of isolate pairs with variances below the overall variance (i.e. < 1) and total isolate number.

Another way to look for evidence of intron invasion/loss is intron sequence divergence. Specifically, vertical inheritance of introns should result in intron divergence being lower when closely-related isolate pairs are compared than when distantly-related isolate pairs are compared, while intron invasion will likely interfere with this pattern. Therefore, as a measure of sequence divergence, we calculated pairwise nucleotide identities for all introns across every pair of isolates and normalized these to the mean identity to account for potential divergence rate differences between introns (supplementary table S6, Supplementary Material online; supplementary Data S3, Supplementary Material online). As we do not necessarily know the correct *Epichloë* phylogeny, we calculated the variance in this normalized pairwise identity across all introns present in both isolates for each isolate pair, under the assumption that if introns are vertically inherited, this variance will be relatively small. We found that most isolate pairs had much smaller variances than the total variance across all introns/isolate pairs, but a small number of introns had high variances, a result that is robust to the exclusion of hybrid isolates (Fig. 5). The highest isolate pair variances (>2.5-fold more than total variance) all involved *E. elymi* or *E. canadensis* and were driven by three divergent introns present in these two isolates (cob_506, cox1_1125 and nad5_717). Redoing this analysis excluding these introns and the other 5 that we found evidence from phylogenetic nesting for possible independent invasion resulted in lower total variance, with ∼75% to 80% of isolate pairs have variances below the total variance level (Fig. 5). Overall, our results are consistent with predominantly vertical inheritance of most introns, although they do not exclude the possibility that independent invasions beyond the 8 candidate cases have also contributed to *Epichloë* intron distribution.

### Lack of Extensive Intron-encoded Gene Degradation Despite Intron Loss

The combination of evidence showing primarily vertical inheritance of introns and the inability of intron distribution to be simply explained by phylogeny suggests there have been a number of intron losses over the course of *Epichloë* evolution, a conclusion that is consistent with the ASR inferences (Table 1). The lifecycle model of Goddard and Burt (1999) predicts that intron loss should be preceded by extensive intron degradation. Therefore, it is surprising that intron nucleotide identities between isolates are high (almost all over 90%; Table 2), suggesting there has not been extensive intron degradation or homing gene loss. The high nucleotide identities could result from strong purifying selection, but this does not appear to be the case as between-isolate amino acid identities are similar to the nucleotide identities (except for nad2_1647, which is lower; Table 2). We thus attempted to more rigorously assess the extent of intron degradation by examining how intact the homing proteins are. We generated alignments of each translated gene encoded in every intron that is present in more than three *Epichloë* isolates (supplementary Data S4, Supplementary Material online). Nearly half of these intron-encoded genes (14/31) show high conservation with little evidence for degradation (such as deletions and frameshifts; supplementary table S7, Supplementary Material online) in any isolate. Even genes that do have deletions and frameshifts are not necessarily loss-of-function mutations, as the frame is only disrupted in a small section with high amino acid identities on both sides of all frameshifts, and most deletions only truncate the 5′ and/or 3′ end (supplementary Information S2, Supplementary Material online; supplementary table S7, Supplementary Material online).

**Table 2:** Intron pairwise nucleotide identities and homing protein amino acid identities between isolates

Intron	Intron pairwise identity range (DNA; mean)^a^	Homing protein pairwise identity range (amino acid)^b^	No. of isolates present in
**1. atp6_521**	98.9 to 100 (99.5)	98.5 to 100	14
**2. atp6_578**	99.7 to 100 (99.8)	99.4 to 100	3
**3. cob_201**	N/A	N/A	1
**4. cob_393**	96.6 to 100 (98.9)	95.2 to 100	28
**5. cob_490**	*^c^	…	30
**6. cob_506**	97.0 to 100 (99.1)	95.8 to 100	32
**7. cob_823**	98.6 to 100 (99.4)	98.4 to 100	21
**8. cox1_50**	99.4 to 99.9 (99.6)	99.1 to 99.7	3
**9. cox1_108**	93.7 to 100 (97.8)	93.3 to 100	10
**10. cox1_199**	98.3 to 100 (99.2)	97.8 to 100	13
**11. cox1_212**	98.9 to 100 (99.6)	97.6 to 100	31
**12. cox1_281**	96.5 to 100 (99.0)	97.2 to 100	31
**13. cox1_615**	99.3 to 100 (99.8)	98.3 to 100	18
**14. cox1_709**	99.4 to 100 (99.7)	98.7 to 100	6
**15. cox1_731**	99.2 to 100 (99.6)	98.4 to 100	9
**16. cox1_867**	99.4 to 100 (99.9)	98.8 to 100	10
**17. cox1_1057**	98.6 to 100 (99.5)	97.6 to 100	27
**18. cox1_1125**	*	…	3
**19. cox1_1262**	95.2 to 100 (99.2)	97.8 to 100	27
**20. cox2_228**	93.7 to 100 (97.5)	91.3 to 100	32
**21. cox2_357**	99.4 to 99.9 (99.6)	99.6 to 100	3
**22. cox2_373**	98.5 to 100 (99.0)	98.0 to 100	12
**23. cox2_651**	98.6 to 100 (99.4)	97.8 to 100	31
**24. cox3_203**	N/A	N/A	1
**25. cox3_216**	*	…	31
**26. cox3_276**	98.8 to 100 (99.5)	98.3 to 100	8
**27. cox3_471**	98.2 to 100 (99.0)	98.4 to 100	16
**28. nad1_144**	98.5 to 100 (99.3)	95.4 to 100	14
**29. nad1_636**	95.9 to 100 (98.2)	95.2 to 100	33
**30. nad2_378**	93.3 to 100 (96.7)	92.0 to 99.8	4
**31. nad2_570**	98.7 to 100 (99.0)	98.7 to 100	5
**32. nad2_1647**	94.7 to 100 (97.5)	83.0 to 100	23
**33. nad4_505**	91.0 to 100 (97.9)	87.2 to 100	9
**34. nad4L_239**	97.0 to 100 (99.1)	95.8 to 100	21
**35. nad5_426**	99.0 to 100 (99.6)	96.6 to 100	15
**36. nad5_570**	98.9 to 100 (99.7)	99.1 to 100	33
**37. nad5_717**	96.8 to 100 (98.6)	§^d^	31
**38. nad6_233**	100	100	2

^a^Range of pairwise nucleotide sequence identities for intron sequences across all isolate pairs where both have the intron.

^b^Range of pairwise amino acid identities for intron-encoding homing proteins across all isolate pairs where both have the intron.

^c^Introns with asterices were excluded as these have a large indel.

^d^Protein alignment was not performed as intron-encoded gene is split into two different ORFs.

We next used protein BLAST searches to confirm that all intron-encoded genes match their host intron type, and to assess (for group I introns) whether potential homing endonuclease motifs are conserved. As expected, all group I intron-encoded homing proteins except one (cob_506, which only matches hypothetical proteins) show similarity to LAGLIDADG or GIY-YIG group I homing endonucleases, and all group II intron-encoded proteins show similarity to reverse transcriptases (supplementary table S7, Supplementary Material online). Examination of the group I intron HEGs showed that for 17 of the 26 introns the LAGLIDADG or GIY-YIG motifs are completely conserved across all isolates carrying the intron, and the motifs are conserved for most isolates in the remaining nine introns (supplementary table S7, Supplementary Material online). We revisited *E. canadensis* to see if loss of homing gene function might explain the failure of some introns to be inherited from its parents (Fig. 3c). Consistent with this hypothesis, we found the *E. elymi* parent is one of two isolates to have a 3′ truncation of cox2_373 (supplementary Information S2, Supplementary Material online). However, the cox3_203 intron appears to have invaded *E. elymi* subsequent to hybridization as this intron is only found in this isolate, and the *E. amarillans* cox1_108 and *E. elymi* cox1_709/cox1_867 homing genes are very similar to those of the other isolates (supplementary Information S2, Supplementary Material online). Thus, intron degradation can only potentially explain the lack of inheritance of one *E. canadensis* intron, and only nine introns (atp6_521, cob_506, cox1_212, cox1_1262, nad1_144, nad1_636, nad2_1647, nad5_570 and nad5_717) have deletions, frameshifts and/or mutations in the HEG motif that are likely to disrupt function in at least one isolate (supplementary Information S2, Supplementary Material online).

Finally, we looked at whether intron loss during *Epichloë* evolution is associated with loss of homing function. To do so, we looked for examples from Fig. 2 where introns appear to have been recently lost and examined whether they are enriched for introns that show evidence for degradation. Of the 13 introns across 8 isolates that appear to represent recent intron loss, only 4 are amongst the 9 introns with evidence for degradation (supplementary Information S3, Supplementary Material online). We also found no evidence for the presence of group II driving intron loss (supplementary Information S4, Supplementary Material online) by providing reverse transcriptase activity that could mediate retro-processing. Thus, we find no evidence for extensive intron-encoded gene degradation and little evidence for any degradation despite the frequent intron loss. This combined with the lack of association between potential homing gene inactivation and intron loss suggest that loss occurs soon after (or possibly even before) loss of homing function.

## Discussion

Here, we document the profile of mtDNA self-splicing introns in the fungal grass endophyte genus, *Epichloë*. These introns, which include both group I and group II introns, occupy 38 different sites in 11 mtDNA protein-coding genes and show substantial presence/absence polymorphism across the genus. For each intron insertion site, all introns found at that site form a monophyletic clade, suggesting that each insertion site has been independently colonized by a separate intron. Our analyses indicate that the presence/absence polymorphism cannot be explained by a simple model of vertical intron inheritance, with ancestral state reconstructions instead inferring multiple intron invasions and losses during evolution of the *Epichloë* genus. These results add to the growing picture of highly variable self-splicing intron presence even amongst closely-related species in the mitochondrial genomes of fungi (Sandor et al. 2018), plants (Mower 2020), and other eukaryotes (Schuster et al. 2017; Guillory et al. 2018; Wideman et al. 2020).

**Fig. 6.**
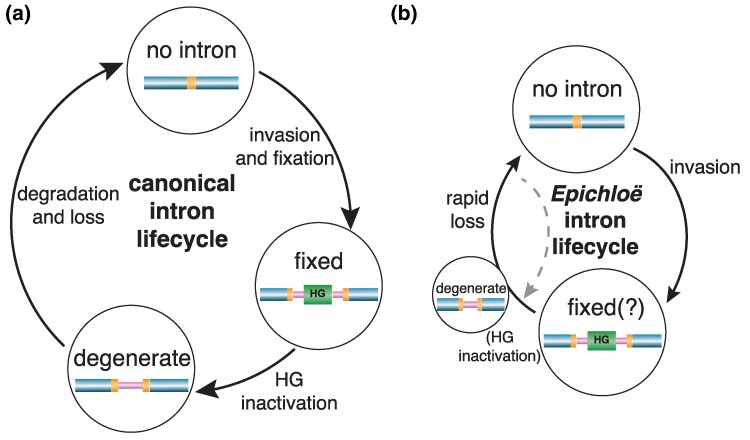
*Epichloë* self-splicing intron lifecycle model. The key difference between the traditional Goddard and Burt (1999) lifecycle model a) and the *Epichloë* model proposed here b) is the *Epichloë* model lacks an extensive intron degradation phase, instead being characterized by rapid intron loss. Rapid loss creates selection pressure for preservation of homing gene (HG) activity (dotted arrow). If loss is sufficiently frequent, intron fixation might not occur and HG degeneration may be limited or absent (indicated schematically by the small degeneration circle). Host gene is indicated in blue, homing endonuclease recognition site in orange, self-splicing intron in pink, and HG in green.

The most striking aspect of our results is that, despite the inference of multiple intron losses, we found almost no evidence for intron degradation. This observation runs counter to the Goddard and Burt intron lifecycle model, which predicts that introns in intermediate stages of degradation should be prominent when losses are observed. Our analyses indicate that the observed intron distributions are not likely to be the consequence of recurrent intron invasions or differences in isolate reproductive histories, but instead that intron loss (alongside predominantly vertical intron inheritance) is the major contributor to *Epichloë* intron distribution. There are 2 potential explanations for intron loss without degradation: (i) that loss occurs soon after homing gene inactivation or (ii) that introns are selectively maintained for long periods before eventual loss. Examples where introns have been maintained because their splicing rate acts as a form of regulation have been observed in other systems (e.g. Rudan et al. 2018; Armaleo and Chiou 2021). However, for this to explain the patterns we observe, most introns would need to confer this benefit, which could not be of a sufficient magnitude to preclude intron loss. More plausibly, selection might maintain a non-homing function in the intron-encoded genes, such as maturases, which facilitate intron splicing (Belfort 2003; Mukhopadhyay and Hausner 2024). Once again, though, this would need to have occurred in most introns and is not consistent with the low levels of nucleotide divergence coupled with similar levels of amino acid divergence that are observed across *Epichloë* introns. More critically, the presence of intact group I intron homing endonuclease motifs in almost all introns and isolates suggest these intron-encoded genes have, at least until recently, encoded functional homing proteins. Thus, we conclude that introns have not been maintained for long periods without functional homing genes, implying that intron loss occurs soon after homing gene inactivation. This suggests that *Epichloë* introns follow an alternative lifecycle to the Goddard & Burt model, one where the length of the homing gene degradation phase is far shorter than in groups such as the Saccharomycetaceae (Goddard and Burt 1999;  Fig. 6).

What could explain the lack of an extensive intron degradation stage in *Epichloë*? The mechanism for intron loss is most likely retroprocessing, although an alternative possibility is a host-encoded mechanism that enzymatically removes introns (Meers et al. 2023). Rapid intron loss might reflect increased rates of the mechanisms underlying retroprocessing, i.e. reverse transcription and/or homologous replacement.

High intron loss rates have other implications for intron dynamics. First, introns might not have time to become fixed in the population before their loss (Fig. 6), a possibility that would require population-level surveys of intron presence/absence to test. Second, high loss rates may mean the homing gene inactivation phase of the lifecycle is limited or skipped completely (Fig. 6), a possibility that is not inconsistent with our results. However, the multicopy nature of the mtDNA means that increasing the rate of intron removal can actually counter intron loss. This is because creation of an intron-less copy of a gene will simply be followed by homing of an intron from another mtDNA genome back into the vacated site. Moreover, because one mtDNA gene can contain several different introns, a retroprocessing event can potentially remove all these introns, creating multiple vacant intron insertion sites that the introns can all then home back into (Yahara et al. 2009). Thus, retroprocessing activity can maintain selection for homing gene function by constantly providing empty sites that can be re-occupied by homing-capable introns. It would be interesting to determine the rates of reverse transcription and homologous replacement in *Epichloë* mtDNA, but this is a major challenge in this group where the ability to genetically manipulate the mtDNA has yet to be established. Instead, modelling approaches could be employed to determine if increased retroprocessing rate is a plausible route for intron loss given the complications highlighted here.

One way to ameliorate the “double-edged sword” effects of retroprocessing and thus promote a short intron degradation phase is suppression of intron homing activity, akin to RNase E activity suppressing group II intron homing (Coros et al. 2008). The fitness costs that self-splicing introns presumably impose on their host genome (Yahara et al. 2009) should create selection pressure for such suppressors to evolve. It is interesting in this regard that we found incomplete inheritance of introns in the hybrid, *E. canadensis*. A more extreme example is found in another *Epichloë* hybrid, which was shown to not inherit any mitochondrial self-splicing introns from one of the parents, although this hybrid's mitochondrial genome also did not undergo any inter-parental recombination (Campbell et al. 2017). While other dynamics such as mitochondrial genome incompatibility could contribute to some of these patterns, incomplete intron inheritance in *Epichloë* hybrids is consistent with suppression of intron homing activity. It has been proposed that competition between intron homing rate and intron cost might lead to a balancing selection dynamic between intron-containing and intron-lacking alleles where neither attains the upper hand (Gogarten and Hilario 2006), and these two alleles can oscillate within populations for long periods of time (Yahara et al. 2009). These models are consistent with our finding of extensive intron presence/absence polymorphism without evidence for high rates of intron invasions from outside of *Epichloë*, and they may also explain the lack of association between sexual status and intron number. Thus, the intron patterns observed in our study might be an outcome of the particular balance between homing rate, intron loss rate, and intron cost in *Epichloë*, with suppressors potentially contributing to this balance.

We found evidence for some independent intron invasions into the mitochondrial genome over the course of *Epichloë* evolution. The most likely source of these introns is related fungi, with similar results having been found for other Cordycipitaceae taxa (Fan et al. 2019). However, how introns can invade *Epichloë* species is not clear, particularly with the endophytic lifestyle of this group. Possibilities include mycoviruses (Myers and James 2022), such as simple mitochondrially-localized *Mitovirus* mycoviruses (Hillman and Cai 2013) or fungal mycoparasites (Naranjo-Ortiz and Gabaldon 2019), but there is currently little evidence for either of these being agents of mitochondrial intron transfer. Abortive hybridization has been proposed previously for horizontal transfer of mitochondrial introns (Marinoni et al. 1999; Koufopanou et al. 2002), but this requires other fungi to contact these endophytic fungi. Thus, as with other systems (e.g. Guillory et al. 2018), the origins of intron invasions are difficult to pin down.

Are the intron dynamics we have characterized here specific to *Epichloë*? We think not, as there is evidence that other groups have similar intron characteristics. For example, all self-splicing introns in the mtDNA protein-coding genes from collections of *Fusarium* (Ponts et al. 2020) and *Aspergillus*/*Penicillium* (Joardar et al. 2012) species have homing genes, suggesting limited homing gene degradation. Furthermore, several studies have shown mitochondrial intron presence/absence polymorphism that does not correlate well with phylogeny (e.g. Brankovics et al. 2018; Guillory et al. 2018; Wideman et al. 2020; Li et al. 2021; Wolters et al. 2023), although whether this is due to intron loss without degradation is not clear. Finally, other taxa within the Cordycipitaceae appear to have had mtDNA intron loss that may not be well correlated with potential homing gene degeneration (Zhang et al. 2015; Fan et al. 2019). Homing gene integrity in mtDNA self-splicing introns is often not carefully assessed, thus a greater emphasis on this feature would help establish whether other taxa show this alternate intron lifecycle characterized by rapid intron loss without extensive homing gene degradation.

In summary, we have characterized striking self-splicing intron dynamism in the *Epichloë* mtDNA genome, with extensive intron presence/absence polymorphism between *Epichloë* isolates. This intron presence/absence is most consistent with vertical inheritance and intron loss, coupled with some independent intron invasions, over the course of *Epichloë* genus evolution. Surprisingly, despite the strong inference of intron losses, we found little evidence for partially degraded introns, in contrast to previous observations and the predictions of the Goddard and Burt self-splicing intron lifecycle model and other models that involve longer intron retention times (Mukhopadhyay and Hausner 2024). Based on these results, we propose the intron lifecycle in *Epichloë* differs from the current model by having a highly abbreviated period of intron degradation as a consequence of frequent retroprocessing events driving intron loss. Frequent intron loss is complicated because it maintains selection for homing activity, and we suggest this may be ameliorated by suppressors of intron homing. We think the intron dynamics found in *Epichloë* are likely to be more widespread, thus more work characterizing intron distributions and activities across different systems will help improve our understanding of the transmission and loss dynamics of these fascinating elements.

## Materials and Methods

### Mitochondrial Genome Sequencing and Assembly

Isolate information and all GenBank accession numbers of sequences reported in this study are presented in supplementary table S1, Supplementary Material online. Genome sequencing of *Epichloë* isolates was performed using Illumina paired-end reads, in some cases supplemented with Oxford Nanopore MinION or PacBio sequencing. Five different assemblers (CLC Genomics Workbench (QIAGEN), Falcon (Chin et al. 2016), GAM (Vicedomini et al. 2013), MaSuRCA (Zimin et al. 2013), and SPAdes (Prjibelski et al. 2020)), each with default parameters, were used to assemble each mitochondrial genome. The assembly with the highest contiguity and recovery of repetitive sequences was then chosen for further analysis (supplementary table S1, Supplementary Material online). Mitochondrial genomes were identified with blastn using the 14 protein-coding genes previously reported in *Epichloë* mitochondrial genomes (Campbell et al. 2017) as queries.

### Sequence Alignment

Sequence alignments were generated using MAFFT (v. 7.490; Katoh and Standley 2013) unless otherwise indicated, and were visualized using Alignment Viewer & Editor (AliView 1.23; Larsson 2014) and Geneious Prime (v. 2022.2.2; https://www.geneious.com).

### Mitochondrial Genome Annotation

Annotations of genes and introns in the mitochondrial genomes was performed using MFannot (http://megasun.bch.umontreal.ca/cgi-bin/mfannot/mfannotInterface.pl) with default parameters (“Genetic code” = 4). Intron-lacking versions of the genes were obtained by performing a blastx search of each protein-coding gene against the GenBank non-redundant protein sequence database using default parameters, and subsequent adjustment of intron-exon boundaries was performed manually based on alignments with these intron-lacking genes.

### Phylogenetic Reconstructions

Intron presence/absence was encoded as binary for dendrogram construction with and without hybrid isolates included. These dendrograms and the phylogenies examining possible intron invasion were constructed using IQ-TREE 2 software (Minh et al. 2020) as follows. First, model selection (Kalyaanamoorthy et al. 2017) was performed as follows:


iqtree2-s{inputfile}

Best models are given in supplementary table S8, Supplementary Material online. Next, rootstrapping (Naser-Khdour et al. 2022) was performed to determine the best root position as follows:


$$\begin{array}{rl} iqtree2 & \;\text{-}s\;\{input\;file\}\text{-}model\text{-}joint\;UNREST\text{-}B\;1000\;\text{-}T\\ & \;AUTO\;\text{-}m\;\{best\;model\} \end{array}$$

Finally, phylogenetic tree construction was performed using the same best model as above, obtaining branch supports with ultrafast bootstrap (Hoang et al. 2018):


iqtree2-s{inputfile}-B1000-TAUTO-m{bestmodel}

To construct *tefA* and *tubB* alignments, only coding sequences were included. To construct the mitochondrial DNA alignments, all introns and the 10 bp on each side of each intron were removed (to avoid potential confounding from co-conversion tracts; Zhang et al. 2015) from the sequences of the 14 primary protein-coding genes (those shown in supplementary fig. S1, Supplementary Material online) for all isolates, and the coding sequences of each isolate concatenated using Alignment Viewer & Editor. Nuclear and mitochondrial DNA phylogenies were constructed using the PhyML maximum likelihood method implemented in NGphylogeny.fr (Lemoine et al. 2019) with the following parameters: proportion of invariant sites was estimated, nucleotide frequencies were empirical, a discrete gamma model was estimated for four categories, the substitution model was general time reversable (GTR), the tree topology search was subtree pruning and regrafting (SPR), and branch support was approximate Bayesian (Anisimova et al. 2011). Phylogenies were visualized using FigTree (http://tree.bio.ed.ac.uk/software/figtree/), including manual placement of the root following rootstrap analysis.

### Ancestral State Reconstruction and Consistency Index Calculation

Ancestral state reconstruction was performed using the Mesquite suite (http://www.mesquiteproject.org) and RASP (Yu et al. 2020), with default parameters. Consistency indices were determined using Mesquite, and the numbers of invasion-only and loss-only events were determined manually from the trees produced by Mesquite.

### *E. canadensis* Hybrid Genome Comparison

The mitochondrial genome section between *nad2* and *nad6* was isolated for *E. canadensis, E. amarillans* and *E. elymi* in Geneious Prime, then aligned (supplementary Data S2, Supplementary Material online). Allocation of parts of the *E. canadensis* genome to each parent was performed in Geneious Prime by manually identifying the parental origin of each *E. canadensis* SNP in the alignment.

### Pan-intron Phylogeny

A multiple sequence alignment of all introns was generated using the ClustalW algorithm in MEGA (Kumar et al. 2018) with the following parameters: gap opening penalty: 10; gap extension penalty: 6.66; DNA rate matrix: IUB; transition rate: 0.5; use negative matrix: off; delay divergent cut off (%): 30. A phylogeny was then constructed from this in MEGA using the following parameters: maximum UPGMA statistical method; test of phylogeny: bootstrap; number of bootstrap replicates: 5000; substitution type: nucleotide; method/model: maximum composite likelihood; substitution to include: transition + transversions; rates among sites: uniform; pattern among lineage: same; missing data treatment: pairwise deletion; select 1st, 2nd, 3rd codon positions, noncoding.

### Relatedness of Closest Intron Blast Matches

The closest matching non-*Epichloë* introns were identified by first generating a consensus sequence from an alignment of each intron or each intron type, then using this as a blastn or Mega-blast (as indicated in supplementary table S5, Supplementary Material online) query with default parameters except the search was restricted to non-*Epichloë* taxa, the non-redundant GenBank database was used, and matches were restricted to the top 10.

For introns with evidence for independent invasions into *Epichloë*, FASTA sequences of the best matches from blast were obtained from Genbank and the orthologous intron extracted by alignment back to the original intron consensus sequence in Geneious Prime. Alignments and phylogenies were then performed as described above.

### Relative Intron Pairwise Divergences

Pairwise distances (nucleotide identities) between isolate pairs were obtained for each intron alignment from Geneious Prime and entered into Excel (supplementary table S6, Supplementary Material online). Relative pairwise distances were calculated for each intron using the formula:


$$\begin{array}{rl} Relative\;pairwise\;distance=Pairwise\;distance/average & \\ pairwise\;distance\;across\;all\;isolate\;pairs \end{array}$$

Relative intron variance was then calculated from all relative pairwise distances across each isolate pair using the VAR.P formula in Excel, relative to the total variance calculated for dataset-wide relative pairwise distances. VAR.P implements the following formula:


$$\frac{\sum {\left(x_{i}-u\right)}^{2}}{n}$$

Where *x_i_* is i-th value in the population, *u* is the population mean, and *n* is the population size.

### Analysis of Intron-encoded Genes

ORFs were annotated in each intron using Geneious Prime (yeast mitochondrial genetic code). The longest ORFs from each intron were extracted and aligned within Geneious Prime (supplementary Data S4, Supplementary Material online). blastp searches using alignment consensus sequences as queries with default parameters were used to confirm that each putative intron-encoded protein matched the intron type. ORF alignments were inspected manually for evidence of degradation and to identify putative group I homing endonuclease motifs.

### Statistical Analyses

All statistical analyses and plots were performed using Prism (v. 9.3.1; Graphpad) unless otherwise noted.

## Supplementary Material

msaf076_Supplementary_Data

## Data Availability

Supplementary Data S1 to S4, Supplementary Material online, containing individual intron alignments; phylogenetic reconstructions, ancestral state reconstructions and the hybrid mitochondrial genome alignment; intron pairwise distances; and aligned translated intron genes, respectively, are available through FigShare (https://doi.org/10.17608/k6.auckland.21988253.; https://doi.org/10.17608/k6.auckland.21988280.; https://doi.org/10.17608/k6.auckland.25869148.; https://doi.org/10.17608/k6.auckland.26170153.).
