# Supplementary material for: An Alternative Self-Splicing Intron Lifecycle Revealed by Dynamic Intron Turnover in Epichloë Endophyte Mitochondrial Genomes
Source: Mol Biol Evol. 2025 Apr 2;42(4):msaf076. doi: 10.1093/molbev/msaf076 (PMC12007492; doi:10.1093/molbev/msaf076)
Supplement: msaf076_Supplementary_Data [file msaf076_supplementary_data.zip › Supplementary_Figure_1 .pdf]

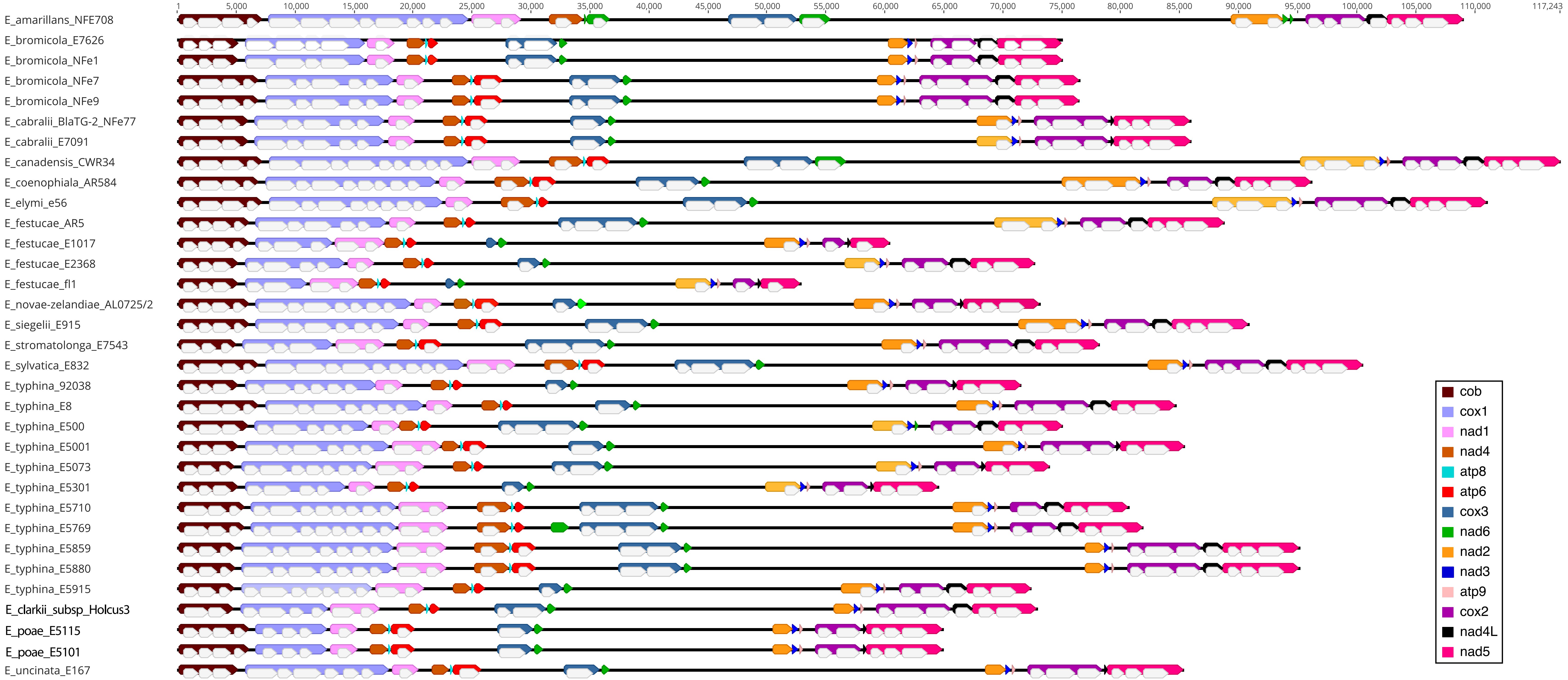

Supplementary Figure 1. Graphical view of the complete mitochondrial genomes of the 33 *Epichloë* isolates used in this study. The protein-coding genes are indicated by the colored block arrows, with the identities of the individual genes shown in the key at bottom right. Self-splicing introns are indicated by the grey block arrows inset into the genes. Size is indicated at top. Other genes, such as tRNA and rRNA genes, are not annotated.
