# Supplementary material for: An Alternative Self-Splicing Intron Lifecycle Revealed by Dynamic Intron Turnover in Epichloë Endophyte Mitochondrial Genomes
Source: Mol Biol Evol. 2025 Apr 2;42(4):msaf076. doi: 10.1093/molbev/msaf076 (PMC12007492; doi:10.1093/molbev/msaf076)
Supplement: msaf076_Supplementary_Data [file msaf076_supplementary_data.zip › Supplementary_Figure_2.pdf]

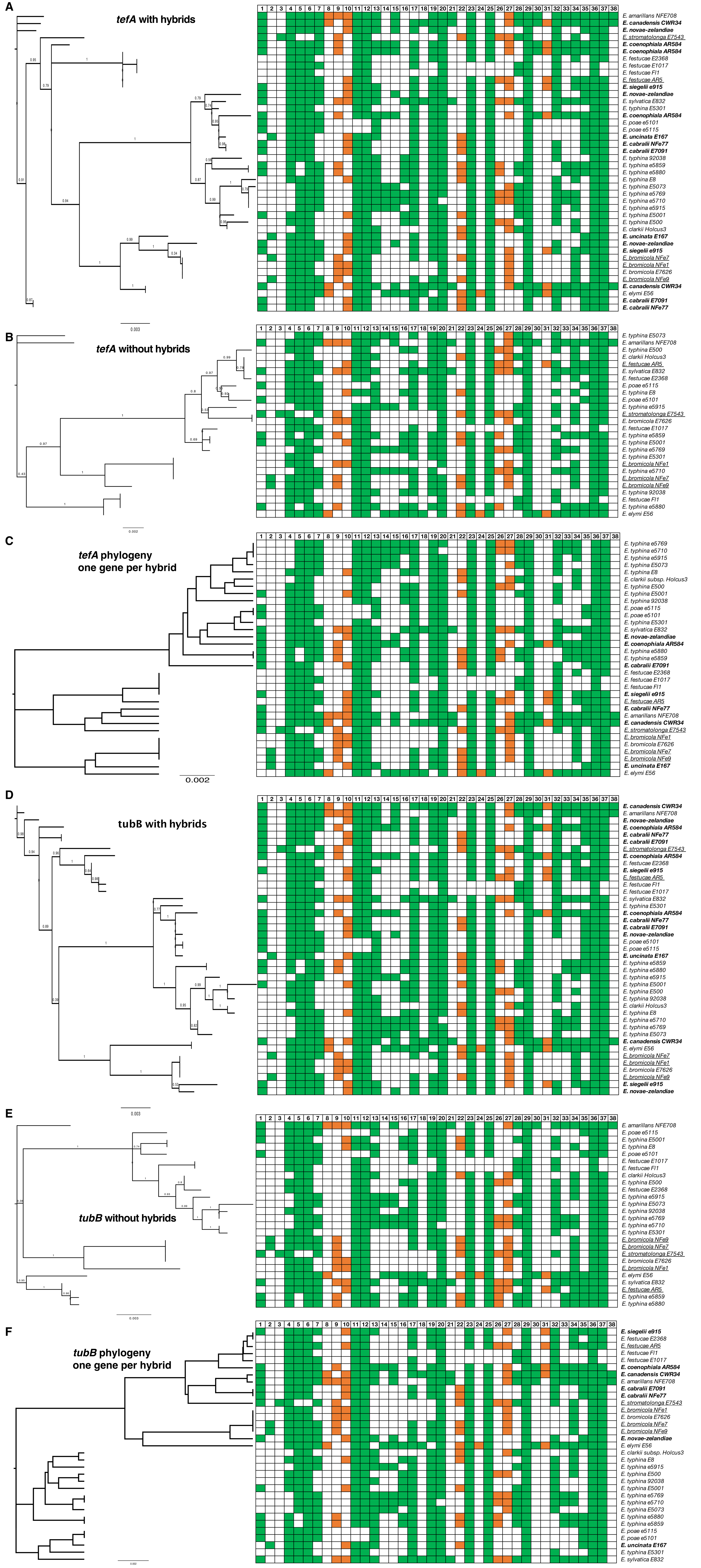

Supplementary Figure 2. Self-splicing intron presence/absence does not correlate with nuclear phylogenies of the *Epichloë* isolates. Phylogenies of the isolates made from partial nuclear *tefA* (**A-C**) and *tubB* (**D-F**) gene sequences, where either all isolates are included (**A,D**), hybrid isolates were excluded (**B,E**), or just one of the two gene copies from hybrid species were included (**C, F**) are shown on the left, alongside the corresponding intron presence/absence matrix to the right. Green is group I intron presence, orange is group II intron presence, white is intron absence. Introns are numbered at top according to **Supplementary Table 2**. Isolate names are shown at far right, with hybrids indicated in **bold** and non-hybrid putative asexual species underlined. Support values are shown for clades where the value is >0.5, and distances are indicated below each phylogeny.
