# Supplementary material for: An Alternative Self-Splicing Intron Lifecycle Revealed by Dynamic Intron Turnover in Epichloë Endophyte Mitochondrial Genomes
Source: Mol Biol Evol. 2025 Apr 2;42(4):msaf076. doi: 10.1093/molbev/msaf076 (PMC12007492; doi:10.1093/molbev/msaf076)
Supplement: msaf076_Supplementary_Data [file msaf076_supplementary_data.zip › Supplementary_Figure_3.pdf]

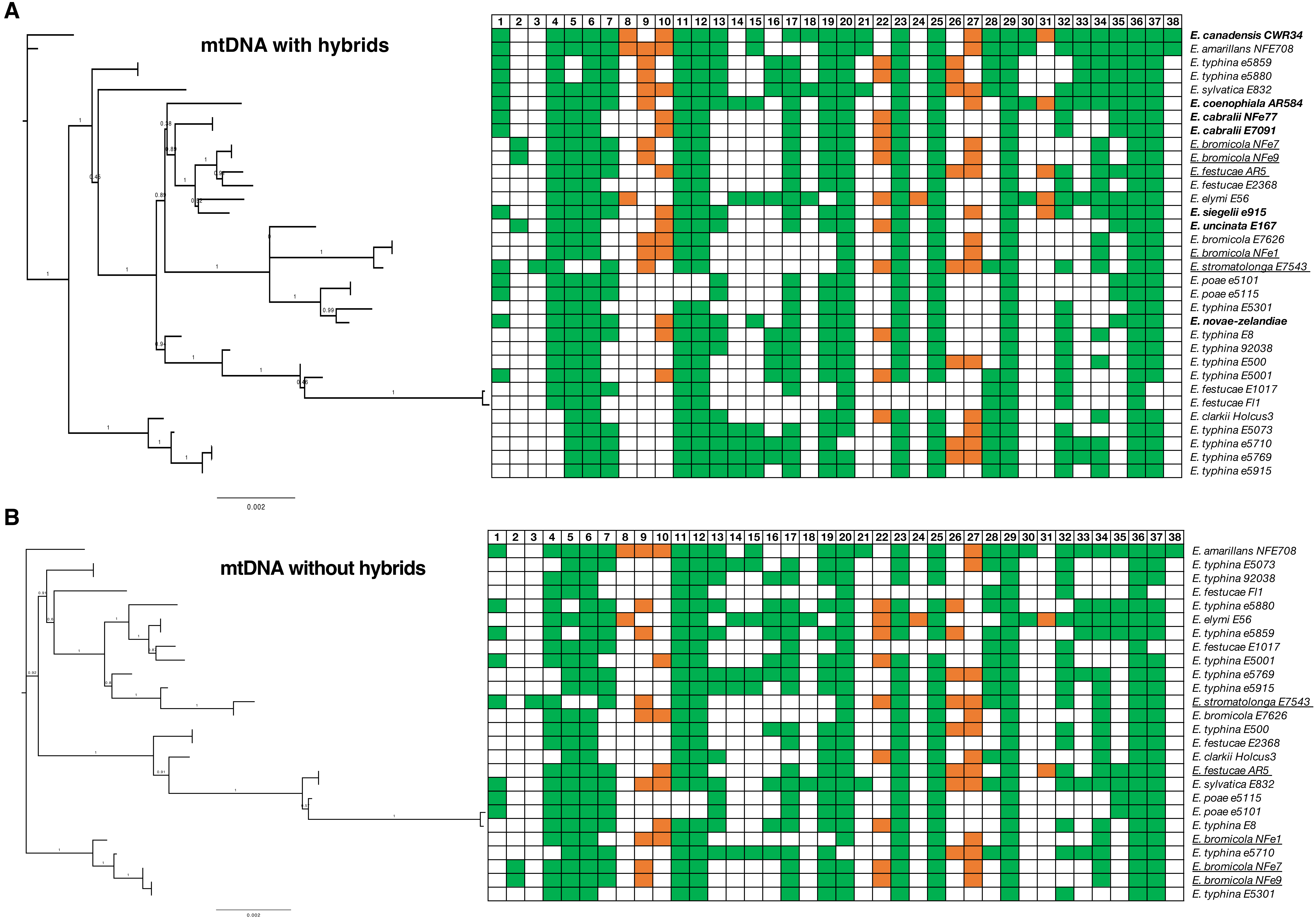

Supplementary Figure X. Self-splicing intron presence/absence does not correlate with mitochondrial phylogenies of the *Epichloë* isolates. Phylogenies of the isolates made from a concatenation of all 14 mitochondrial protein-coding gene sequences after removal of introns and the 10 bp each side of each intron, where either all isolates are included (**A**) or where hybrid isolates were excluded (**B**), are shown on the left, alongside the corresponding intron presence/absence matrix to the right. Green is group I intron presence, orange is group II intron presence, white is intron absence. Introns are numbered at top according to **Supplementary Table 2**. Isolate names are shown at far right, with hybrids indicated in **bold** and non-hybrid putative asexual species underlined. Support values are shown for clades where the value is >0.5, and distances are indicated below each phylogeny.
