# Supplementary material for: An Alternative Self-Splicing Intron Lifecycle Revealed by Dynamic Intron Turnover in Epichloë Endophyte Mitochondrial Genomes
Source: Mol Biol Evol. 2025 Apr 2;42(4):msaf076. doi: 10.1093/molbev/msaf076 (PMC12007492; doi:10.1093/molbev/msaf076)
Supplement: msaf076_Supplementary_Data [file msaf076_supplementary_data.zip › Supplementary_Figure_4.pdf]

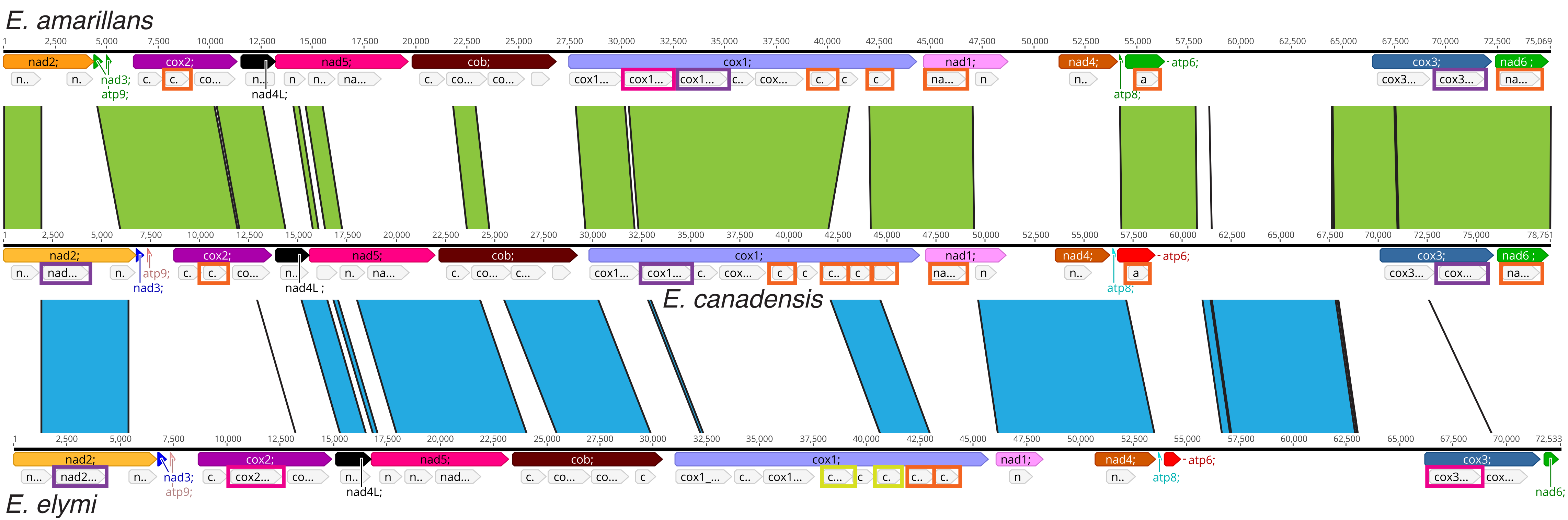

Supplementary Figure 4. The mitochondrial genome of the hybrid, *E. canadensis*, is a mosaic of its two parental genomes. Maps of the *E. canadensis* (middle) and its parents (*E. amarillans* at top and *E. elymi* at bottom) mitochondrial genomes are shown, with protein-coding genes annotated in colours and self-splicing introns in grey below the gene annotations. Only the part of the mitochondrial genome containing the 14 protein-coding genes examined in this study are depicted. The parental origins of blocks of the *E. canadensis* genome are indicated by colored boxes (green = *E. amarillans* origin; blue = *E. elymi* origin), as determined by identifying the parental type of every SNP/intron in *E. canadensis*. Borders between blocks are approximate because SNP density is typically low. Single lines represent cases where just a single SNP is from that parent with both flanking SNPs being from the other parent (these could be homoplasies). Coloured boxes around introns indicate group I introns present in one parent and the hybrid (orange) or one parent only (yellow), and group II introns present in one parent and the hybrid (purple) or one parent only (pink). Cutting induced in a parental mitochondrial genome by group I introns present only in the other parental genome can explain some recombination blocks, but not all. For example, the recombination blocks around the *cox1* and *nad1* genes could be explained by DSBs triggered by introns in this region that are only present in one genome. In contrast, the recombination blocks around the *nad4L*, *nad5*, and *cob* genes are not associated with intron polymorphisms.
