# Supplementary material for: An Alternative Self-Splicing Intron Lifecycle Revealed by Dynamic Intron Turnover in Epichloë Endophyte Mitochondrial Genomes
Source: Mol Biol Evol. 2025 Apr 2;42(4):msaf076. doi: 10.1093/molbev/msaf076 (PMC12007492; doi:10.1093/molbev/msaf076)

Supplementary Information 1. Examining the evidence from diverged introns for intron invasion

For all phylogenetic reconstructions here, the IQ-TREE 2 software was used to first determine the best model and then for rootstrapping analysis. Phylogenetic reconstruction was subsequently performed using the best model with ultrafast bootstrapping, and the root was set to the highest-scoring position from the rootstrapping analysis.

cob_490

There are two intron variants that vary primarily by an insertion. The closest blastn match to the long one (present in 16 isolates) is to the *Purpureocillium atypicola* mitochondrial genome (PP812219.1). The closest blastn match to the short one (present in 14 isolates) is to the *Paraisaria heteropoda* mitochondrial genome (NC_081046.1). The insertion was trimmed to a single nucleotide before phylogenetic reconstruction, including both closest matches from the blastn. Both *P. atypicola* and *P. heteropoda* introns nest within a clade containing many *Epichloë* isolates, suggesting there might have been independent insertions of this intron. However, both non-*Epichloë* introns are on long branches, although the rootstrap has high support (>90%).

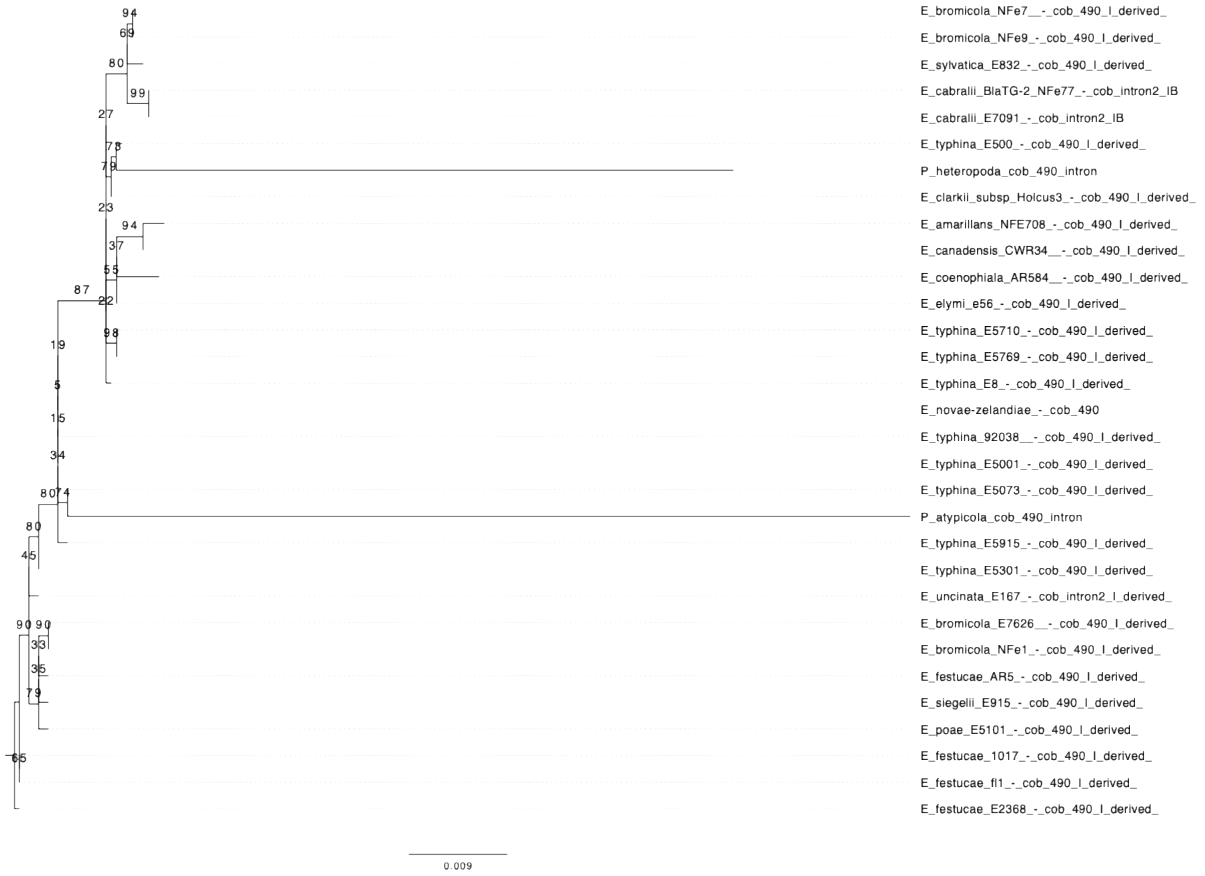

Note: *E. poae* isolate E5115 was omitted from this tree as the IQ-TREES 2 software was unable to construct a bootstrapped tree with all isolates included. However, the intron sequence for this isolate is 100% identical to that of *E. poae* E5101.

cob_506

There are two intron variants that vary primarily by an insertion that is present in 3 isolates, with 29 isolates not having this insertion. For both intron types the closest blastn match is to the *Ustilaginoidea virens* mitochondrial genome (CP135112.1). The *U. virens* intron sits outside of a clade containing all *Epichloë* isolates, thus there is no evidence for independent introductions of this intron. The rootstrap has high support (>90%).

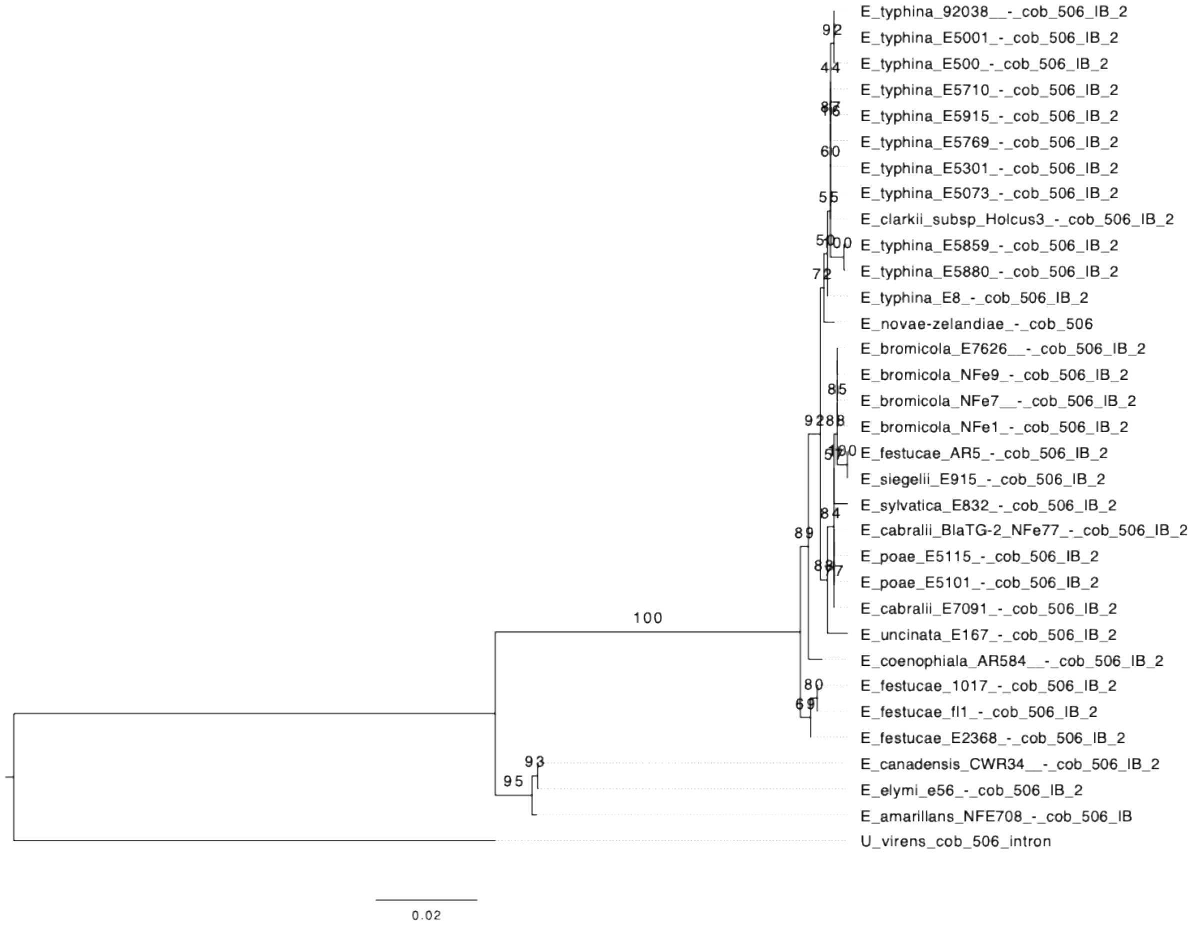

cox1_108

One isolate (*E. amarillans*) has a somewhat diverged intron sequence compared to the other *Epichloë* introns. For both intron types the closest blastn match is to the *Paraisaria gracilloides* mitochondrial genome (NC072283.1). The *P. gracilloides* and *E. amarillans* introns sit in a separate clade to the rest of the *Epichloë* isolates. This could possibly indicate evidence for independent introductions of this intron, with an unknown intron being the source for the non-*amarillans* introns, although the rootstrap support is weak (37.3%).

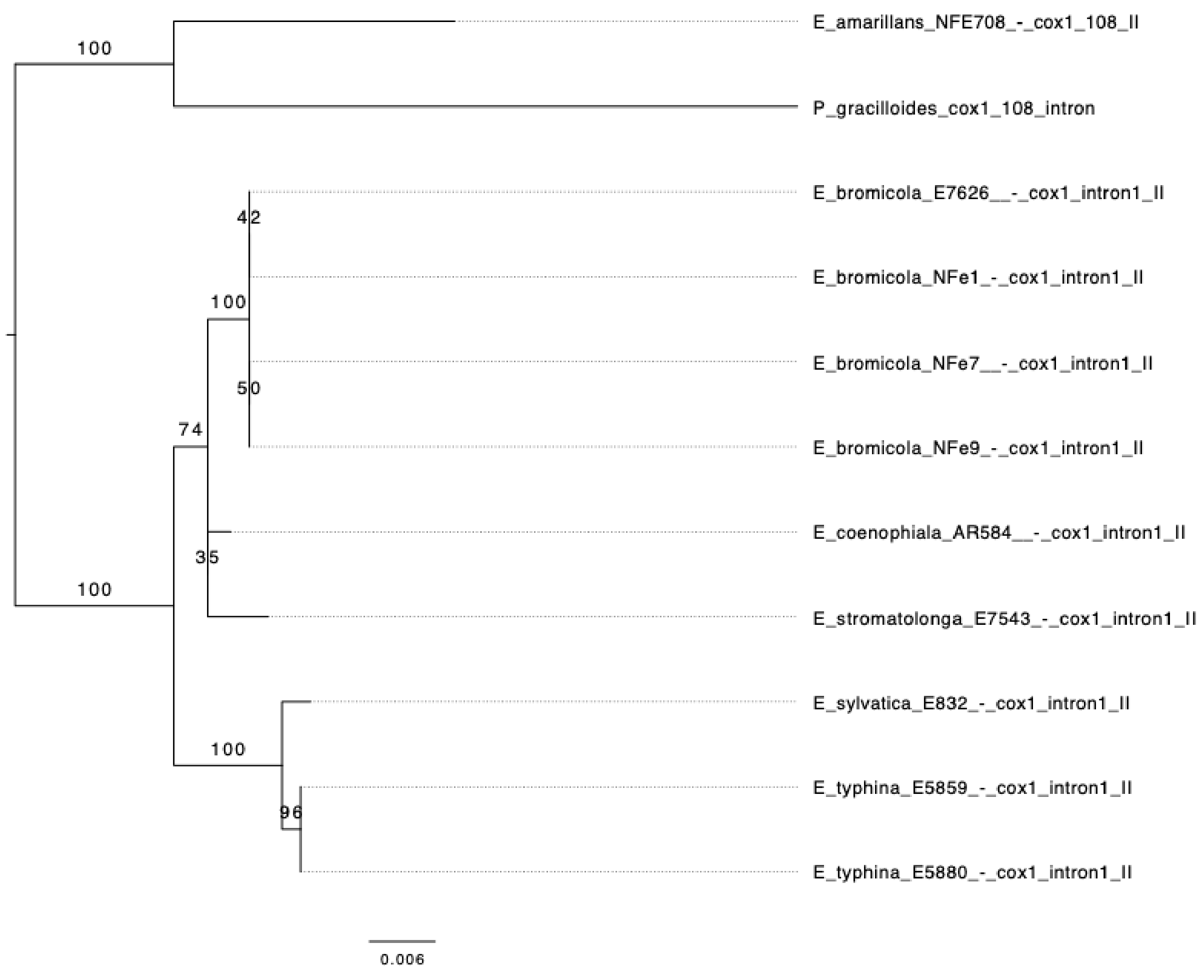

cox1_1125

One isolate (*E. sylvatica*) has a somewhat diverged intron sequence compared to the other two isolates (*E. elymi* and *E. canadensis*). For the intron from the latter two isolates, the closest blastn match is to the *Ustilaginoidea virens* mitochondrial genome (CP135112.1). For the *E. sylvatica* intron, the closest blastn match is to the *Purpureocillium atypicola* mitochondrial genome (PP812219.1). The *E. elymi* and *E. canadensis* introns form a clade with *U. virens*, and the *P. atypicola* and *E. sylvatica* introns sit outside of this clade, suggesting there have been two independent introductions of this intron. The rootstrap support is moderate (66.2%).

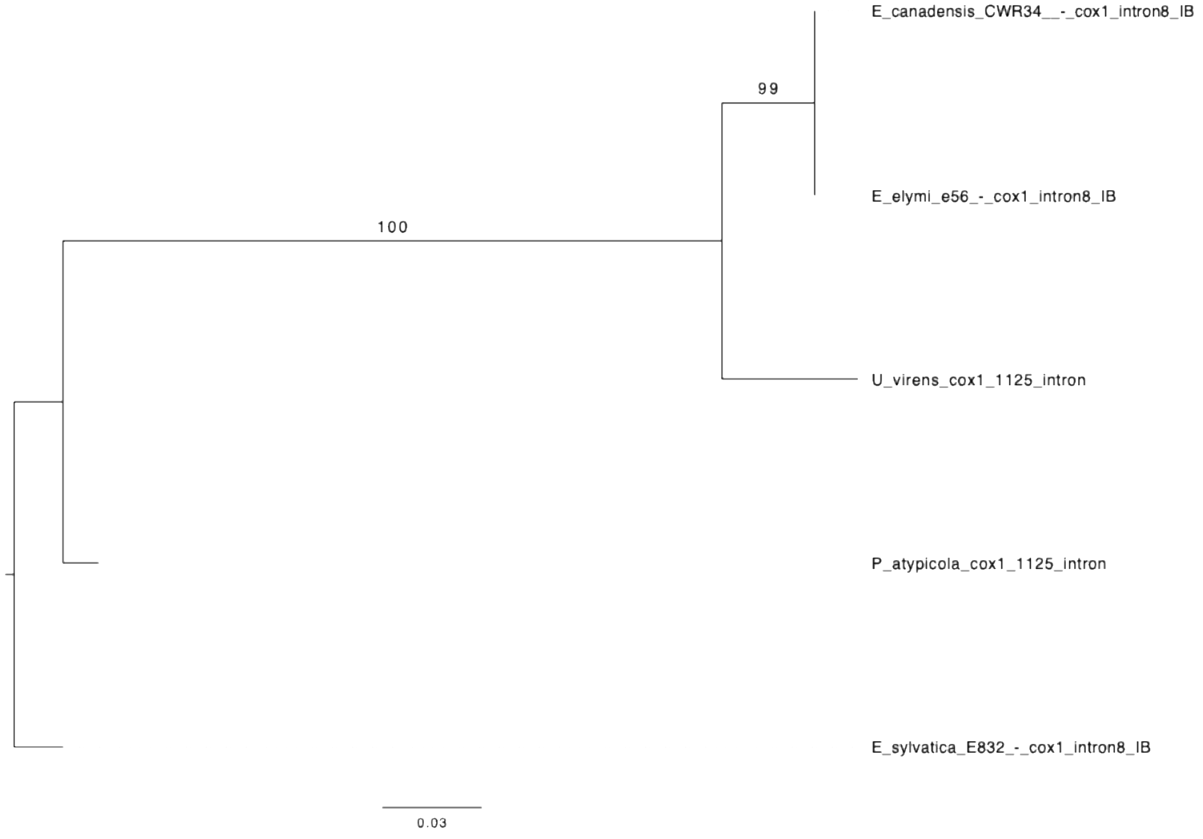

cox1_1262

One isolate (*E. festucae* E2368) differs from the other *Epichloë* introns in that is has a small deletion. For both intron types the closest blastn match is to the *Purpureocillium atypicola* mitochondrial genome (PP812219.1). The *P. atypicola* intron sits outside a clade with all *Epichloë* introns (note that *Tolypocladium ophioglossoides* was also included in this tree as this is the most closely-related species from the blastn analysis, and this also sits outside of the *Epichloë* clade). Thus, there is no evidence for independent introductions of this intron. The rootstrap support is weak (52.9%).

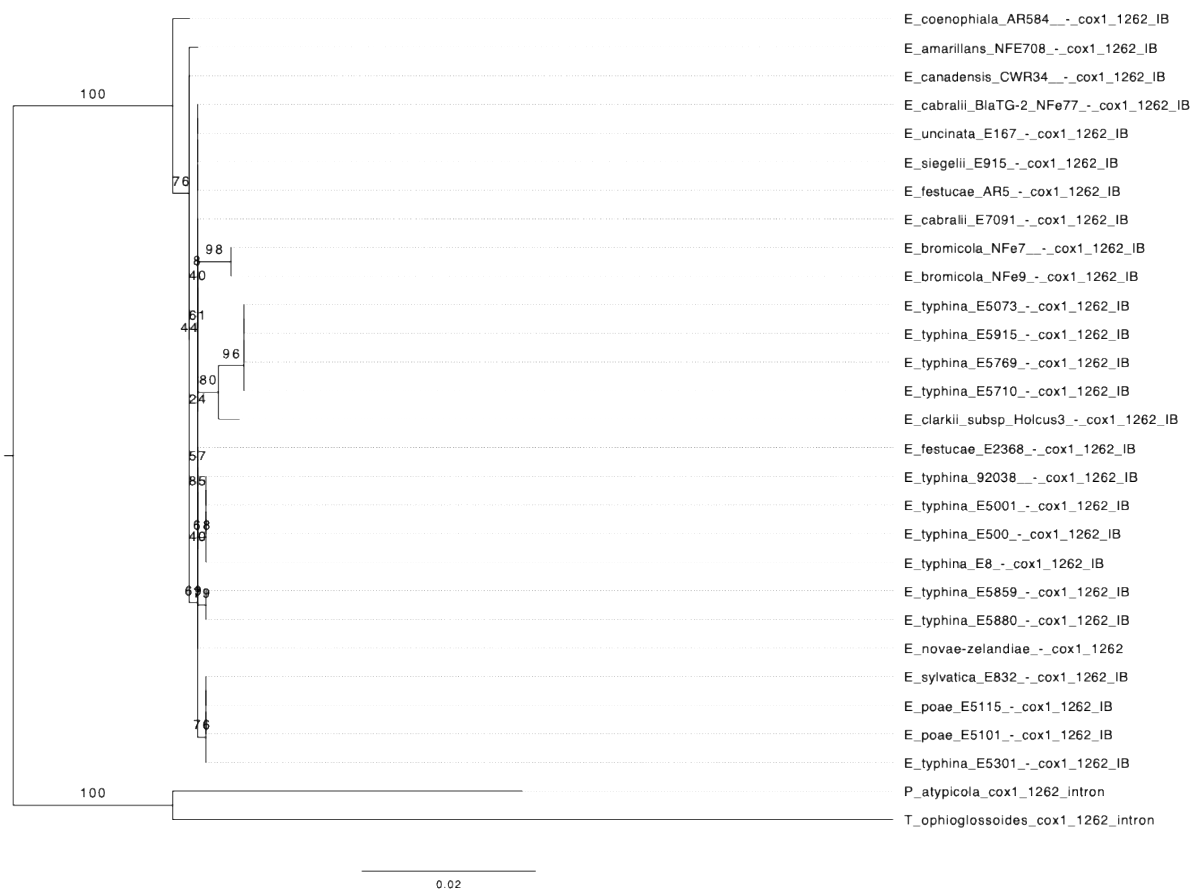

cox2_228

No isolate is particularly diverged compared to the others, and the closest blastn match is to the *Tolypocladium guangdongense* mitochondrial genome (NC054274.1). The *T. guangdongense* intron sits outside a clade with all *Epichloë* introns. Thus, there is no evidence for independent introductions of this intron. The rootstrap support is moderate (76.5%).

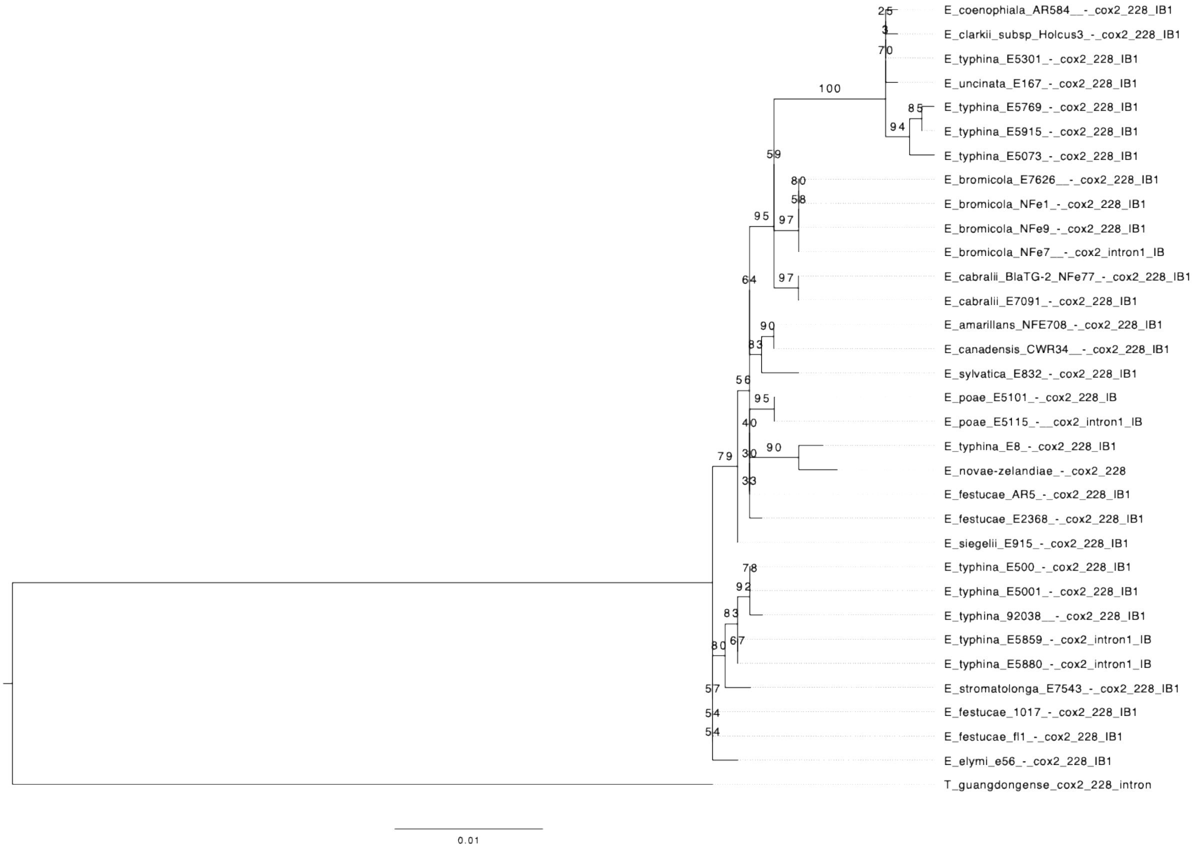

cox3_216

There are two introns, shared by approximately half the isolates each. For both intron types the closest blastn match is to the *Purpureocillium atypicola* mitochondrial genome (PP812219.1). The *P. atypicola* intron sits inside a clade with all *Epichloë* introns (note that *Metarhizium pinghaense* was also included in this tree as this is the most closely-related species from the blastn analysis, but this sits outside of the *Epichloë* clade) in the tree with the short type, but on a long branch. In contrast, this *P. atypicola* intron sits outside the clade with all *Epichloë* introns in the tree with the long intron type (as does the *M. pinghaense* intron). Thus, there is some evidence for independent introductions of this intron. The rootstrap support is strong (99% for the short tree and 63.3% for the long tree).

Short intron type:

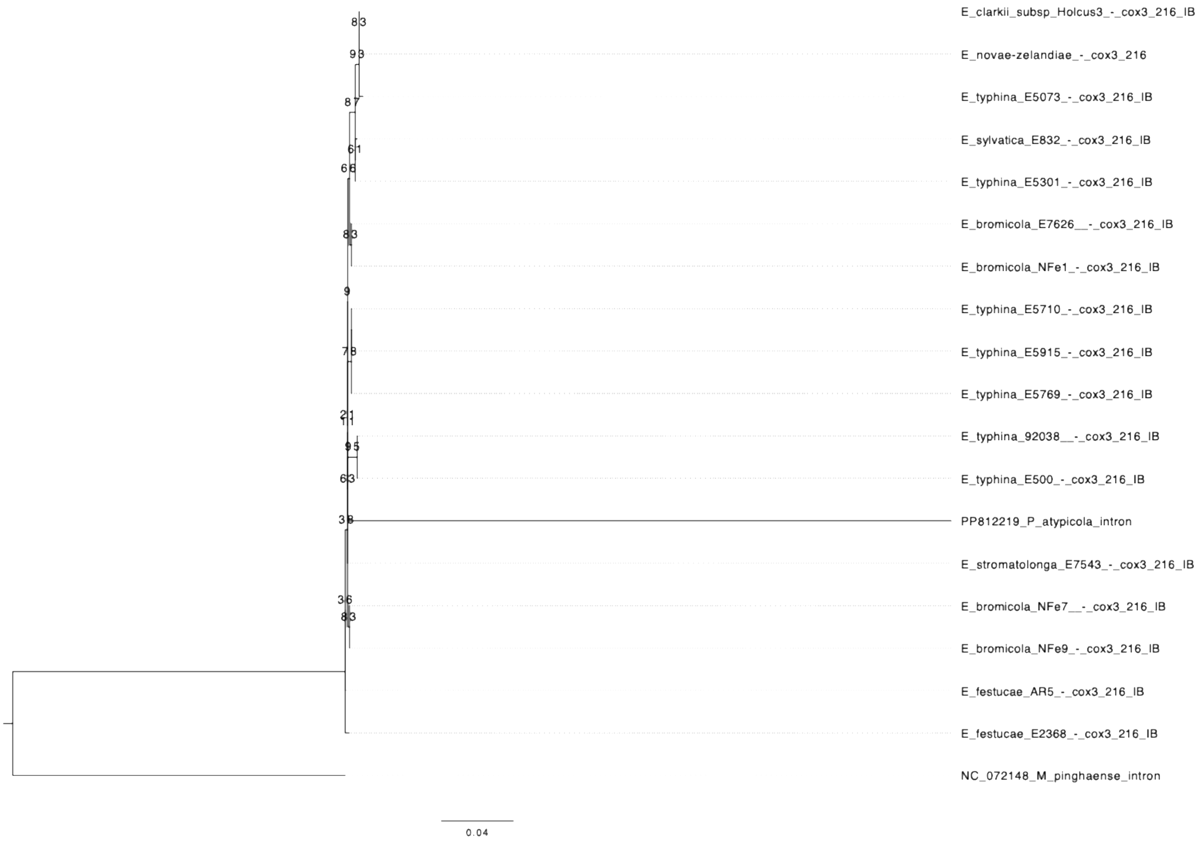

Long intron type:

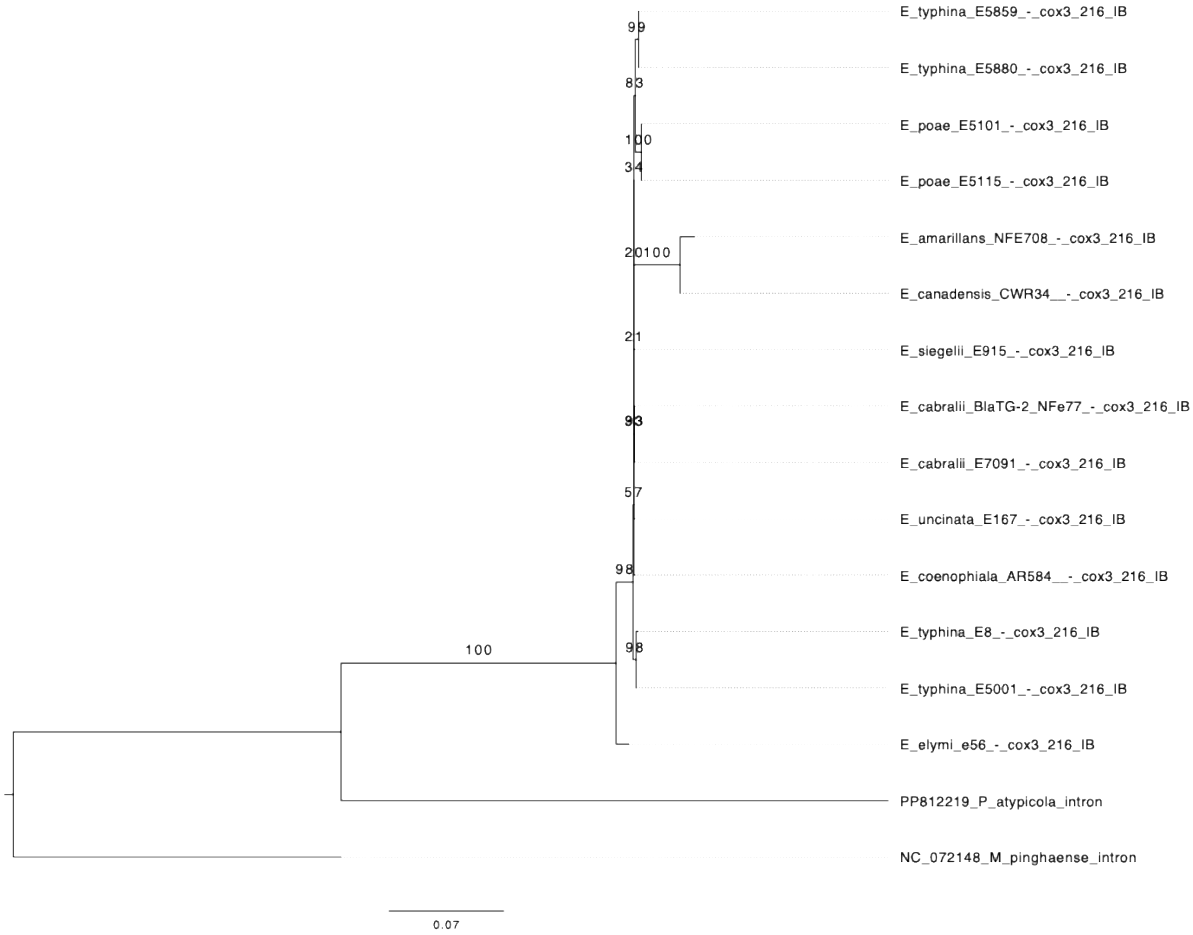

nad1_636

No isolate is particularly diverged compared to the others, and the closest blastn match is to the *Paraisaria gracilloides* mitochondrial genome (NC_072283.1). The *P. gracilloides* intron sits outside a clade with all *Epichloë* introns (note that *Metarhizium pinghaense* was also included in this tree as this is the most closely-related species from the blastn analysis, and this also sits outside of the *Epichloë* clade). Thus, there is no evidence for independent introductions of this intron. The rootstrap support is strong (84.9%).

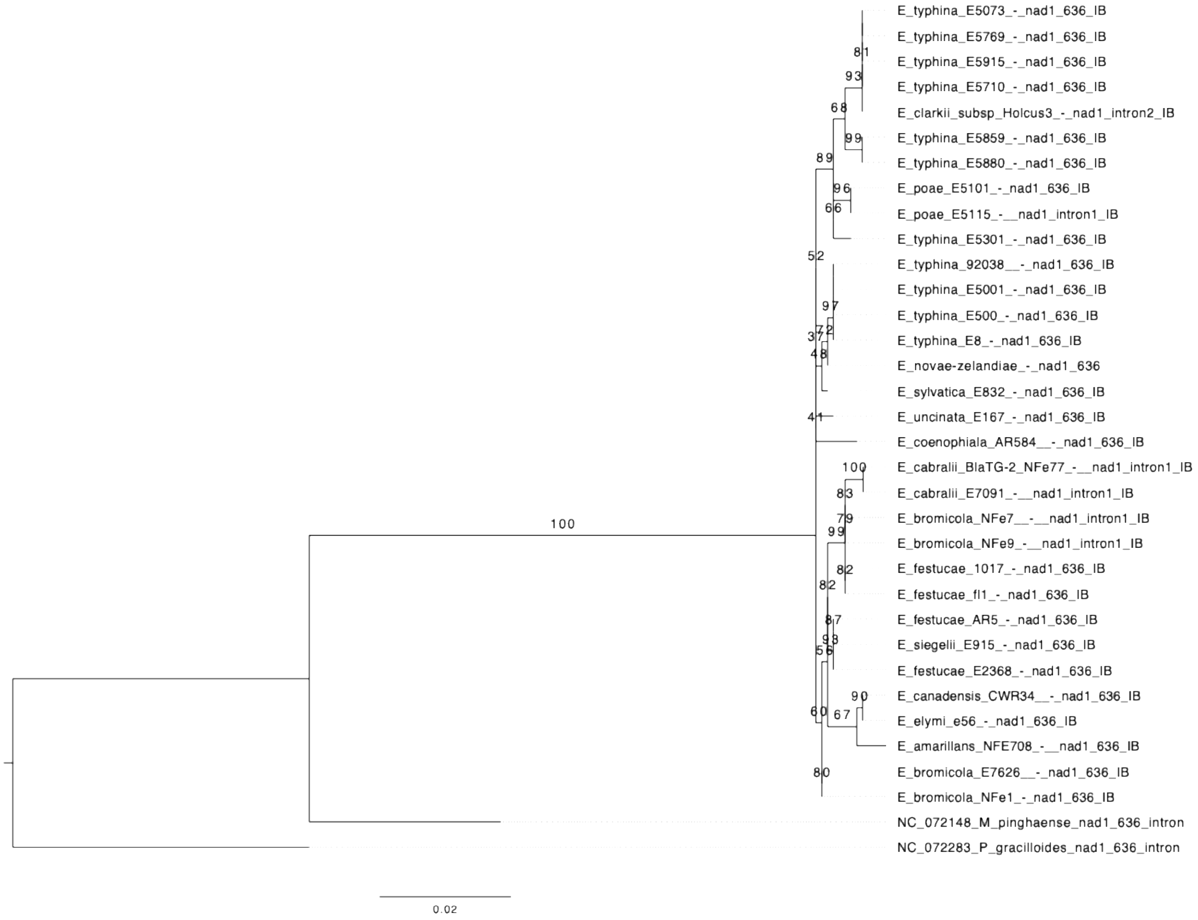

nad2_378

One isolate (*E. coenophiala*) has a somewhat diverged intron sequence compared to the other *Epichloë* introns. For both intron types the closest blastn match is to the *Trichoderma cornu-damae* mitochondrial genome (MW525445.1). The *T. cornu-damae* intron sits outside of a clade that contains all the *Epichloë* introns (note that *Metarhizium album* was also included in this tree as this is the most closely-related species from the blastn analysis, and this also sits outside of the *Epichloë* clade). Thus, there is no evidence for independent introductions of this intron. The rootstrap support is moderate (68.6%).

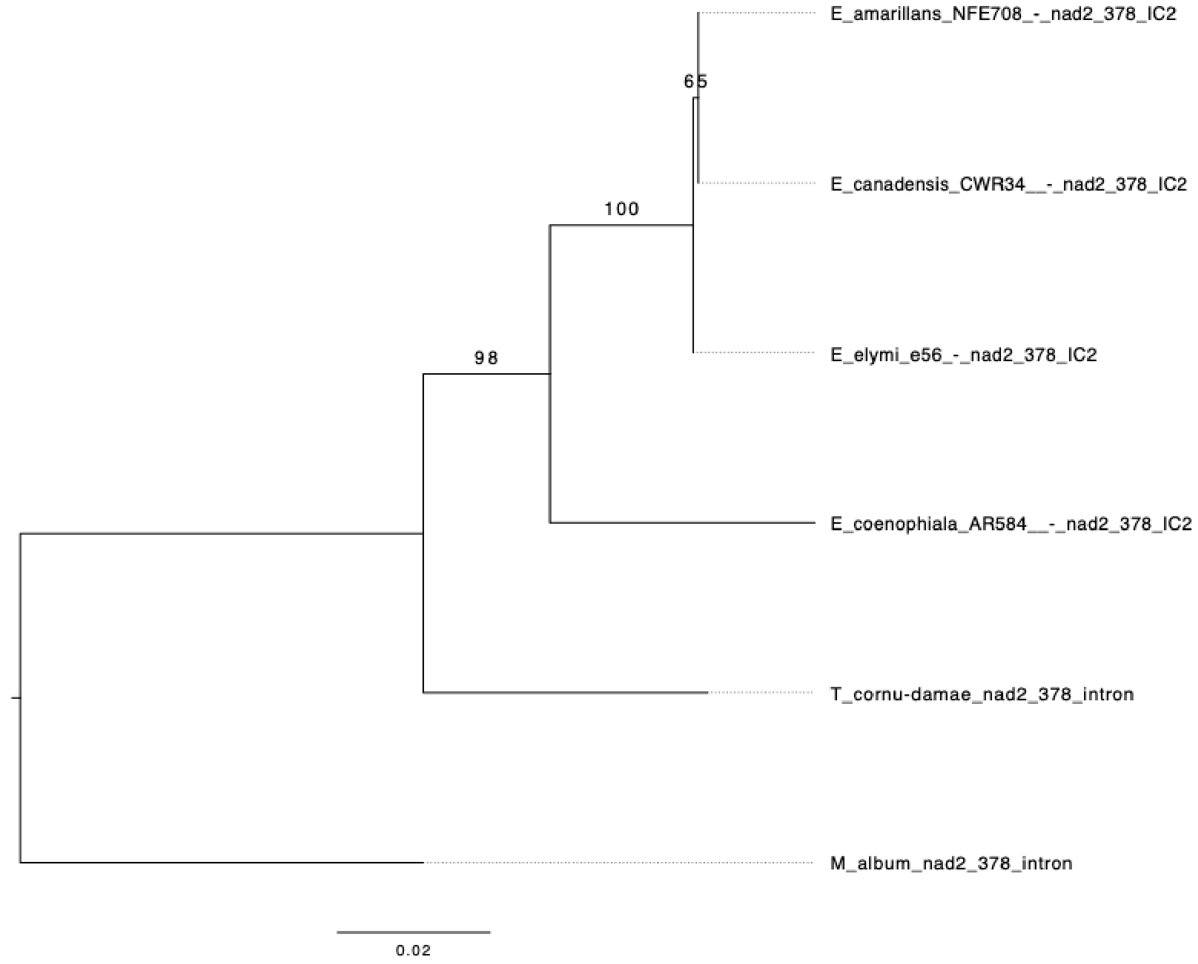

nad2_1647

No isolate is particularly diverged compared to the others, and the closest blastn match is to the *Tolypocladium guangdongense* mitochondrial genome (NC054274.1). The *T. guangdongense* intron sits inside a clade with other *Epichloë* introns, suggesting there might have been independent introductions of this intron. However, the *T. guangdongense* intron is on a long branch and the rootstrap support is very weak (23.5%).

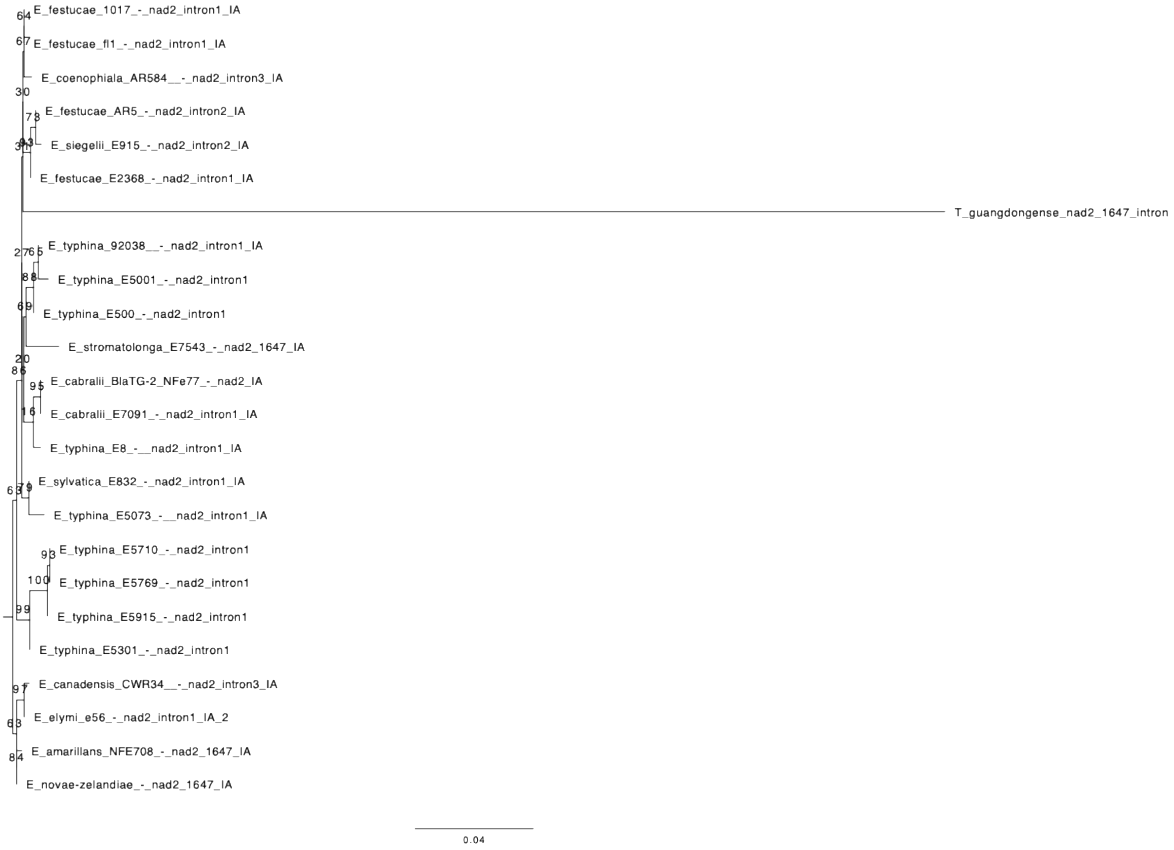

nad4_505

One isolate (*E. coenophiala*) has a somewhat diverged intron sequence compared to the other *Epichloë* introns. For both intron types the closest blastn match is to the *Metarhizium album* mitochondrial genome (NC_057480.1). The *M. album* and *E. coenophiala* introns form a clade that nests within other *Epichloë* introns, suggesting there might have been independent insertions of this intron. However, the *M. album/E. coenophiala* clade is on a long branch and the rootstrap support is weak (40.9%).

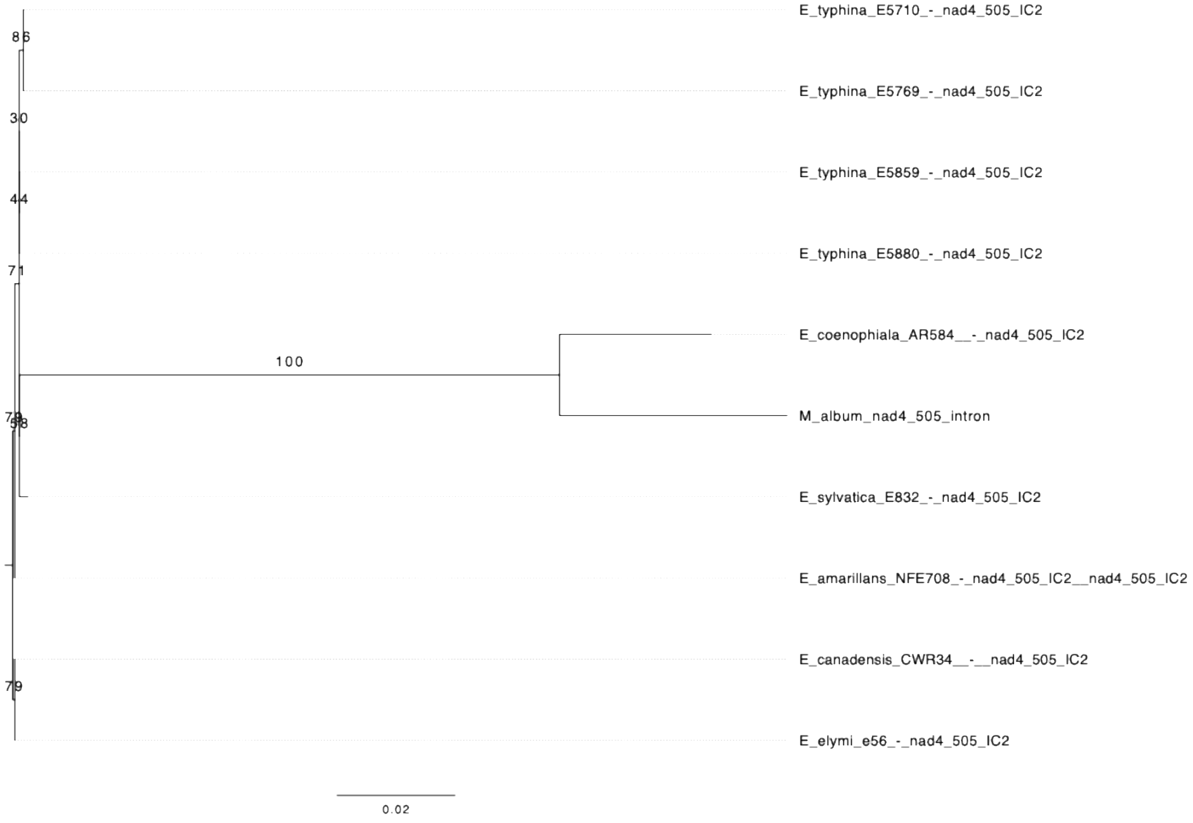

nad5_717

Two isolates (*E. canadensis* and *E. elymi*) have somewhat diverged intron sequences compared to the other *Epichloë* introns. For both intron types the closest blastn match is to the *Metarhizium rileyii* mitochondrial genome (NC_047289.1). The *M. rileyii* intron forms a clade together with the *E. canadensis* and *E. elymi* introns that is nested within other *Epichloë* isolates, suggesting there might have been independent insertions of this intron. However, the *M. album/E. canadensis/E. elymi* introns are on long branches, and the *E. canadensis* and *E. elymi* introns both have a substitution in the HEG motif, opening the possibility that both are inactive and therefore undergoing accelerated evolution. Moreover, the rootstrap support is weak (49.9%).

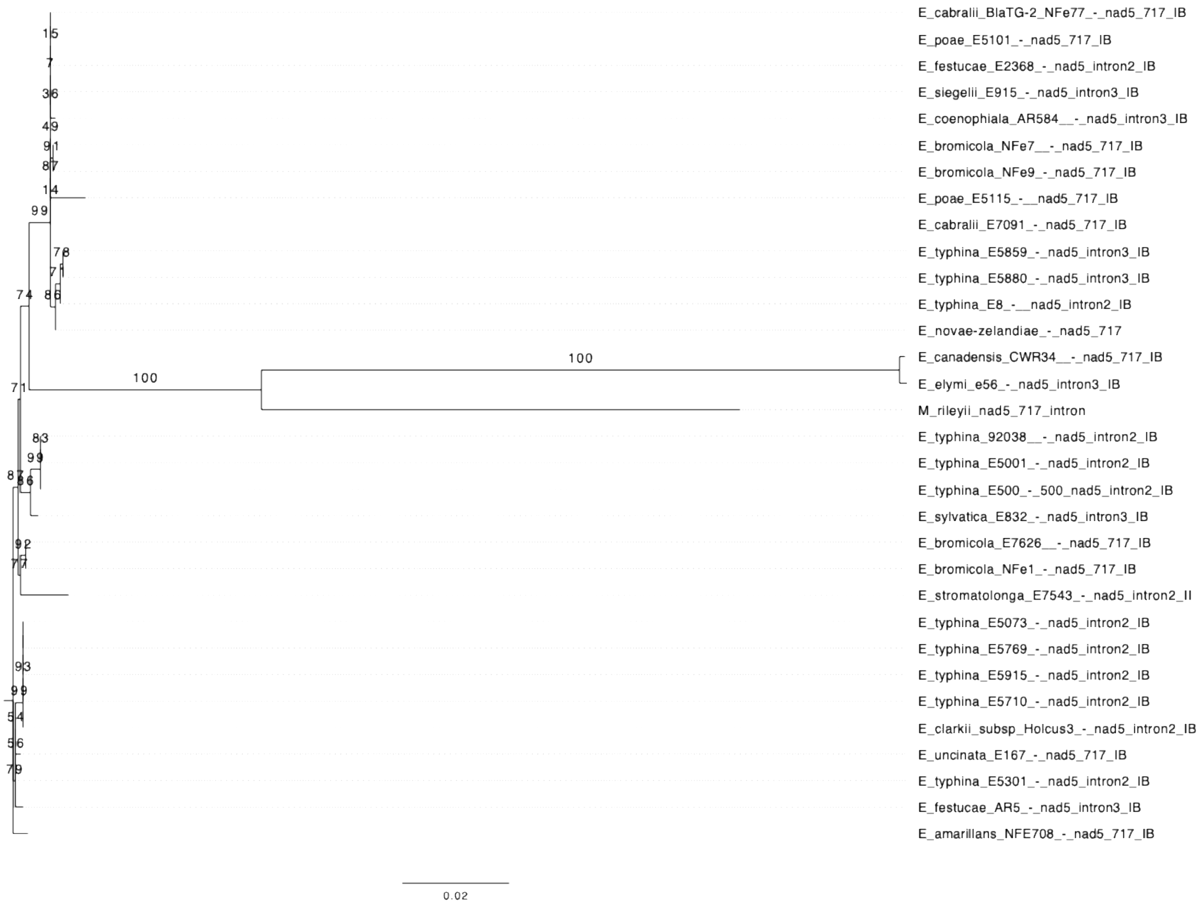

Supplement: msaf076_Supplementary_Data [file msaf076_supplementary_data.zip › Supplementary_information_1.pdf]
