# Supplementary material for: An Alternative Self-Splicing Intron Lifecycle Revealed by Dynamic Intron Turnover in Epichloë Endophyte Mitochondrial Genomes
Source: Mol Biol Evol. 2025 Apr 2;42(4):msaf076. doi: 10.1093/molbev/msaf076 (PMC12007492; doi:10.1093/molbev/msaf076)
Supplement: msaf076_Supplementary_Data [file msaf076_supplementary_data.zip › Supplementary_information_2.pdf]

Supplementary Information 2 – investigation of intron degradation for introns present in more than three isolates

atp6_521
Most full length, but E5859 and E5880 have a truncation at the 5' end, and NFe77 and E7091 have a frameshift for the last ~1/3rd of the protein, the sequence of which is conserved after the frameshift. Consensus matches GIY-YIG endonucleases by DELTA-BLAST protein – best match is GIY-YIG endonuclease [Hirsutella minnesotensis] YP_009160660.1 (fungus; Ophiocordycipitaceae). The GIY-YIG domain is conserved in all except the two isolates with 5' truncations, in which it is missing.

cob_393
Most sequences same, but e56, AR5, E2368, E167 and Fl1 have a frameshift for the last ~1/3rd of the protein. In all cases the sequence past the frameshift is conserved. Fl1 also has a ~50 aa 5' truncation. Other than the region of the frameshift, the aa sequences are very similar. Consensus matches GIY-YIG endonucleases by DELTA-BLAST protein – best non-Epichloe match is GIY-YIG endonuclease, partial [Tolypocladium cylindrosporum] YP_009750543.1 (fungus, Ophiocordycipitaceae). Based on match to GIY-YIG endonuclease [Fusarium acuminatum CS5907] CDL73465.1, the GIY-YIG domain is conserved in all isolates.

cob_490
There appears to be an insertion of ~120 aa into the ORF for ~1/2 of the isolates. Both versions have very similar aa sequences within each version between isolates. Similarly, between the two versions the sequence that is shared (~200 aa) shows very high identity between isolates. Consensus of the ORF with the insertion matches LAGLIDADG endonucleases by DELTA-BLAST protein – best non-Epichloe match is GIY-YIG endonuclease, partial [Ophiocordyceps sp.] UED14375.1 (fungus, Ophiocordycipitaceae). This has two potential LAGLIDADG motifs, one of which is in the shared region and one in the inserted region. The former is conserved across all isolates. Consensus of the ORF without the insertion matches the same LAGLIDADG endonucleases by DELTA-BLAST protein, but only in the shared region. The LAGLIDADG motif in the non-shared region is also conserved across all isolates that contain this region.

cob_506
Most sequences same, but e56, E. amarillans and E. canadensis have a ~90 aa insertion compared to the rest. The insertion does not disrupt the aa sequence much, as the insertion is a duplication at both the nucleotide and aa sequence levels. AR584 has a ~45 aa truncation at the 5' end. Otherwise, the protein sequences are well conserved across all isolates. DELTA-BLAST protein only detects hypothetical proteins from fungi, the closest non-Epichloe match is hypothetical protein [Metarhizium rileyi] YP_009763302.1 (fungus; Clavicipitaceae).

cob_823
Sequences all nearly identical. Consensus matches LAGLIDADG endonucleases by DELTA-BLAST protein – best match is hypothetical protein [Calonectria ilicicola] QIJ45901.1 (fungus; Hypocreales). The putative LAGLIDADG motif has a W=>L substitution in E5710, E5769 and E5915.

cox1_108
Group II intron. All isolates very conserved in protein sequence. E5859 and E5880 have ~60 aa truncations at the 3’ end. Consensus matches group II intron reverse transcriptase/maturases by DELTA-BLAST protein, with best match being reverse transcriptase domain-containing protein, partial [Golovinomyces cichoracearum] YP_010119255.1 (fungus; Erysiphales).

cox1_199
Group II intron. All isolates very conserved in protein sequence, except AR5 has ~100 aa truncation at the 3’ end. Consensus mostly matches some reverse transcriptases by DELTA-BLAST protein, with the best non-Epichloe match being reverse transcriptase domain-containing protein, partial [Erysiphe pisi] YP_010119171.1 (fungus; Erysiphales).

cox1_212
Sequences all nearly identical, except that E. novae-zelandia has a ~20 aa truncation at the 5’ end, and E. amarillans is missing most (the first ~210 of ~340 aa) of the ORF. Consensus matches GIY-YIG endonucleases by DELTA-BLAST protein – best non-Epichloe match is GIY-YIG endonuclease, partial [Drechmeria coniospora] KAH8836195.1 (fungus; Ophiocordycipitaceae). The putative GIY-YIG motif is completely conserved in all isolates except E. amarillans for which this whole part is missing. Possibly intron degradation in E. amarillans (only).

cox1_281
There are two ORFs, corresponding to ~370 aa and ~285 aa proteins. The first protein is highly conserved, although E. elymi, E. canadensis and E. amarillans all have a ~12 aa insertion about 1/3rd of the way through the protein. E. novae-zelandiae, E. canadensis and E. amarillans also have a ~15 aa truncation at the 5’ end. Consensus matches hypothetical proteins from fungi, including with close matches, by DELTA-BLAST protein, and the closest match is hypothetical protein, partial [Metarhizium rileyi] YP_009763322.1 (fungus; Clavicipitaceae).
The second protein is also highly conserved, with only E2368 showing lack of conservation in the first ~35 aa. Consensus matches LAGLIDADG endonucleases by DELTA-BLAST protein – best non-Epichloe match is LAGLIDADG endonuclease [Cordyceps militaris] UFJ44006.1 (fungus; Cordycipitaceae). One putative LAGLIDADG motif is conserved in everything except E2368, which is totally different, while the other is completely conserved except for E. uncinata which has a single aa substitution in the middle of the motif, and some polymorphism between L and I in several isolates also in the middle of the motif.

cox1_615
All isolates with very similar protein sequences. Consensus matches LAGLIDADG endonucleases by DELTA-BLAST protein – best non-Epichloe match is LAGLIDADG endonuclease, partial [Hirsutella thompsonii] YP_009546960.1 (fungus; Ophiocordycipitaceae). The two putative LAGLIDADG motifs are conserved across all isolates.

cox1_709

All isolates with very similar protein sequences. Consensus matches LAGLIDADG endonucleases and similar proteins by DELTA-BLAST protein – best match is hypothetical protein J6816_mgp14, partial [Tolypocladium guangdongense] YP_010044477.1 (fungus, Ophiocordycipitaceae). The putative LAGLIDADG motif is conserved across all isolates.

cox1_731
All isolates with very similar protein sequences. Consensus matches LAGLIDADG endonucleases and similar proteins by DELTA-BLAST protein – best match is LAGLIDADG endonuclease, partial [Tolypocladium cylindrosporum] YP_009750546.1 (fungus, Ophiocordycipitaceae). The putative LAGLIDADG motif is conserved across all isolates.

cox1_867
All isolates with very similar protein sequences. Consensus matches LAGLIDADG endonucleases and similar proteins by DELTA-BLAST protein – best match is LAGLIDADG endonuclease, partial [Cladobotryum mycophilum] YP_010043378.1 (fungus; Hypocreales). The putative LAGLIDADG motif is conserved across all isolates.

cox1_1057
All isolates show very similar aa sequences. Consensus matches GIY-YIG endonucleases by DELTA-BLAST protein – best match is GIY-YIG endonuclease, partial [Hirsutella minnesotensis] YP_009160652.1 (fungus; Ophiocordycipitaceae). The putative GIY-YIG motif is conserved across all isolates.

cox1_1262
All isolates show similar aa sequences, except E2368 which has a 5' truncation of ~60 aa, and E. novae-zelandiae and E. amarillans which have ~10 aa 5' truncations. Consensus matches GIY-YIG endonucleases by DELTA-BLAST protein – best match is hypothetical protein, partial [Fusarium temperatum] AKM98023.1 (fungus; Hypocreales). The putative GIY-YIG motif is conserved across all isolates, except that E2368 is missing the first part of the motif because of the 5' truncation.

cox2_228
All isolates show similar aa sequences. There is some variability around where the gene starts, which may reflect ORF calling rather than polymorphism in the HEG itself. There are two polymorphic indels around the middle of the gene, each shared between a number of isolates. Also, some minor polymorphism around where the gene stops. Consensus matches GIY-YIG endonucleases by DELTA-BLAST protein – best non-Epichloe match is hypothetical protein J6816_mgp20, partial [Tolypocladium guangdongense] YP_010044471.1 (fungus, Ophiocordycipitaceae). The putative GIY-YIG motif is conserved across all isolates.

cox2_373
Group II intron. All isolates show similar aa sequences, although E. elymi and E. uncinata have ~60 aa truncations at the 3' end. Consensus matches group II intron reverse transcriptase/maturases by DELTA-BLAST protein, with best non-Epichloe match being reverse transcriptase domain-containing protein, partial [Schizosaccharomyces pombe] QDP17161.1 (fungus; Schizosaccharomycetales).

cox2_651
Most isolates show very similar protein sequences, but E. amarillans, E915, NFe77 and E7091 have a frameshift ~1/3rd of the way through the ORF. The aa sequence is conserved on either side of the frameshift. There is also a lot of variation of the termination at the 3' end, with 7 isolates (all but E. amarillans not overlapping with the frameshift isolates) terminating ~35- 60 aa before the main termination site. Consensus matches GIY-YIG endonucleases by DELTA-BLAST protein – best match is hypothetical protein [Trichoderma cornu-damae] QVV23918.1 (fungus; Hypocreales). The putative GIY-YIG motif is conserved across all isolates, and is a little upstream of the frameshift site.

cox3_216
There are two different ORFs present. All 31 isolates have the first ORF, while 14 of these isolates also have a second ORF 3' to the first one. It looks like this intron has picked up a second HEG in some isolates.
For the first ORF, there are a number of frameshifts, with 5 isolates having 1 frameshift and another 4 having two frameshifts, and these frameshifts occurring at 5 different positions across the ORF. However, the aa sequences are fairly well conserved across all isolates, including before and after the frameshift positions. There is some polymorphism between isolates encompassing ~30 aa as to where the ORF starts. Otherwise, all isolates have full-length sequences (including all frameshifts). Consensus matches LAGLIDADG endonucleases by DELTA-BLAST protein – best match is LAGLIDADG endonuclease, partial [Metarhizium rileyi] YP_009763337.1 (fungus; Clavicipitaceae). The putative LAGLIDADG motif is conserved across all isolates, except for E5073 that has a single aa substitution.
For the second ORF, the sequence is highly conserved between all isolates, except E. elymi which has a frameshift ~2/3rds of the way through the protein. Consensus matches LAGLIDADG endonucleases by DELTA-BLAST protein – best match is LAGLIDADG endonuclease [Cordyceps militaris] UFJ43970.1 (fungus; Cordycipitaceae). There are two putative LAGLIDADG motifs and both are conserved across all isolates.

cox3_276
Group II intron. All isolates show very similar aa sequences. Consensus matches group II intron reverse transcriptase/maturases by DELTA-BLAST protein, with best non-Epichloe match being hypothetical protein [Ophiocordyceps sp.] UED14394.1 (fungus; Ophiocordycipitaceae).

cox3_471
Group II intron. All isolates show very similar aa sequences. Consensus matches group II intron reverse transcriptase/maturases by DELTA-BLAST protein, with best non-Epichloe match being group II intron reverse transcriptase/maturase [Juglanconis juglandina] ATI20532.1 (fungus; Diaporthales).

nad1_144
There is a lot of variability at the 5' end, with 6 isolates having a frameshift about ¼ of the way through the protein, although the aa sequence is more-or-less conserved either side of this. E1017 instead is truncated for this 5' part. However, it is possible the gene actually starts where E1017 starts/frameshift occurs, although the matches to other proteins (below) are across the whole ORF. In addition, E1017 and E. amarillans have ~55 aa

truncations at the 3’ end. Consensus matches GIY-YIG endonucleases by DELTA-BLAST protein – best match is GIY-YIG endonuclease, partial [Chrysoporthe austroafricana] YP_009262069.1 (fungus; Diaporthales). The putative GIY-YIG motif is conserved across all isolates.

nad1_636
Very variable. 1017, Fl1, 5859, 5880, NFe7 and NFe9 have a 5’ ~65 aa stretch that is absent from the others. Many of the remaining isolates have their ORF start around here, but 8 isolates only start a further ~60 aa downstream. In addition, E. sylvatica, 5001, 5115, NFe7 and NFe9 have frameshifts about 60% of the way along the full-length protein. E. amarillans, E. elymi and E. canadensis have ~45 aa truncations at the 3’ end. The combination of starting late and truncating early mean that E. elymi and E. canadensis are only ~70 aa in length. Otherwise, the aa sequences are reasonably well conserved between isolates, including either side of the frameshifts. Consensus matches GIY-YIG endonucleases by DELTA-BLAST protein – best match is GIY-YIG endonuclease [Hirsutella thompsonii] YP_009546965.1 (fungus; Ophiocordycipitaceae). The putative GIY-YIG motif is at the 5’ end of the ORF, and thus is only present in 1017, Fl1, 5859, 5880, NFe7 and NFe9, but the first half of the motif does not match the actual motif and seems unlikely to be real. In addition, all isolates have a reverse strand ORF near middle of intron. This is almost completely conserved across all isolates. However, this is only 84 aa in length, and doesn’t match anything by DELTA-BLAST protein.

nad2_378
All isolates show similar aa sequences. Consensus matches LAGLIDADG endonucleases by DELTA-BLAST protein – best match is hypothetical protein [Trichoderma cornu-damae] QVV23910.1 (fungus; Hypocreales). The putative LAGLIDADG motif is conserved across all isolates.

nad2_570
Group II intron. All isolates show very similar aa sequences. Consensus matches include reverse transcriptases by DELTA-BLAST protein – best non-Epichloe match is hypothetical protein [Ophiocordyceps lanpingensis] UEX92732.1 (fungus; Ophiocordycipitaceae).

nad2_1647
The aa sequences are quite variable. Most isolates start at approximately the same point, except E. elymi and E. canadensis which have a ~60 aa truncation at the 5’ end. Many of the isolates have one or two frameshifts within the protein. The position of this is quite variable between isolates – at least three different sites. Consensus matches LAGLIDADG endonucleases by DELTA-BLAST protein – best match is NADH dehydrogenase subunit 2 [Tolypocladium guangdongense] YP_010044467.1. The putative LAGLIDADG motif is conserved across all isolates, except the two with the 5’ truncation for which it is completely missing.

nad4L_239
All isolates show similar aa sequences, except E. sylvatica has a frameshift ~1/3rd into the protein, but the aa sequence is conserved either side of the frameshift. Furthermore, there is a 3 aa indel polymorphism shared by ~1/2 the isolates each. Consensus matches

LAGLIDADG endonucleases by DELTA-BLAST protein – best match is NADH dehydrogenase subunit 4L [Trichoderma asperellum] YP_009466109.1 (fungus; Hypocreales). The putative LAGLIDADG motif is conserved across all isolates.

nad4_505
All isolates show very similar aa sequences. Consensus matches LAGLIDADG endonucleases by DELTA-BLAST protein – best match is LAGLIDADG endonuclease, partial [Metarhizium album] YP_010164170.1 (fungus; Clavicipitaceae). The possible LAGLIDADG motif is conserved across all isolates.

nad5_426
Most isolates show very similar aa sequences, but E. sylvatica, E5859, and E5880 have a stop codon and a ~20 aa deletion ~1/3rd through the protein, with the aa sequence conserved either side of this. These isolates, along with E. amarillans, start ~20 aa further upstream than the other isolates. Consensus matches LAGLIDADG endonucleases by DELTA-BLAST protein – best match is LAGLIDADG endonuclease, partial [Chrysoporthe deuterocubensis] YP_009262101.1 (fungus; Diaporthales). There are two possible LAGLIDADG motifs, both of which are conserved across all isolates.

nad5_570
All isolates show very similar aa sequences, except Fl1 which has two stop codons with ~40 aa and ~20 aa deletions at ~1/3rd and ¾ through the protein, with the remaining parts being conserved. Consensus matches LAGLIDADG endonucleases by DELTA-BLAST protein – best non-Epichloe match is LAGLIDADG endonuclease, partial [Cordyceps pruinosa] QQA36258.1 (fungus; Cordycipitaceae). There are two possible LAGLIDADG motifs, both of which are conserved across all isolates, including Fl1.

nad5_717
Quite variable. All isolates have a similar starting point, except E. stromatolonga which has a ~60 aa truncation at the 5' end. There are a large number of frameshifts in many isolates, with three prominent frameshifted sites. Consensus matches LAGLIDADG endonucleases by DELTA-BLAST protein at the 5' half of the ORF – best non-Epichloe match is LAGLIDADG endonuclease [Metarhizium rileyi] YP_009763312.1 (fungus; Clavicipitaceae). There are no particular matches to the 3' half of the ORF outside Epichloe. Therefore, this is probably two ORFs, rather than a frameshift. Of the two possible LAGLIDADG motifs, both are conserved across all isolates except E. elymi and E. canadensis which have a single aa substitution at position 2 in each case, and the first motif which is part of the E. stromatolonga truncation.

There is some evidence for 7 introns being degraded in at least one isolate, other than some variability of the 5' and 3' ends: atp6_521, cox1_212, cox1_1262, nad1_637, nad2_1647, nad5_570 and nad5_717. Of these, atp6_521, cox1_212, cox1_1262 and nad2_1647 have possible loss of the functional HEG motif in a small number of isolates, and nad1_636 is highly variable between isolates with the possibility that a number are non-functional, nad5_570 has one isolate with frameshifts but with conserved putative HEF motifs, and nad5_717 has a number of isolates with frameshifts and a few isolates with potential degradation of their HEG motif.
