# Supplementary material for: An Alternative Self-Splicing Intron Lifecycle Revealed by Dynamic Intron Turnover in Epichloë Endophyte Mitochondrial Genomes
Source: Mol Biol Evol. 2025 Apr 2;42(4):msaf076. doi: 10.1093/molbev/msaf076 (PMC12007492; doi:10.1093/molbev/msaf076)
Supplement: msaf076_Supplementary_Data [file msaf076_supplementary_data.zip › Supplementary_information_3.pdf]

Supplementary Information 3. Isolates/introns with evidence for recent loss

Manual inspection of the phylogenetically-arranged presence/absence matrices from **Figure 2** showed potential recent intron losses, as inferred by the presence of the missing intron amongst close relatives, of thirteen introns:
cob_393
cox1_212
cox1_281
cox1_867
cox1_1057
cox1_1262
cox2_228
cox2_651
cox3_216
nad2_570
nad2_1647
nad4L_239
nad5_717

from eight isolates:
E. amarillans
E. festucae E1017 and Fl1
E. poae e5101 and e5115
E. typhina E5073 and e5710
E. clarkii

Only introns/isolates where recent losses were inferred from all four phylogenies in **Figure 2** were selected.

Of these thirteen introns, only four (cox1_212, cox1_1262, nad2_1647, and nad5_717) were found to have potential intron-encoded homing gene degradation in at least one isolate (**see Supplementary Data X**). Therefore, intron degradation does not appear to predict recent intron loss in these data.
