# Supplementary material for: An Alternative Self-Splicing Intron Lifecycle Revealed by Dynamic Intron Turnover in Epichloë Endophyte Mitochondrial Genomes
Source: Mol Biol Evol. 2025 Apr 2;42(4):msaf076. doi: 10.1093/molbev/msaf076 (PMC12007492; doi:10.1093/molbev/msaf076)
Supplement: msaf076_Supplementary_Data [file msaf076_supplementary_data.zip › Supplementary_information_4.pdf]

Supplementary Information 4. Group I intron loss does not appear to be facilitated by the presence of group II intron reverse transcriptases

The intron-encoded protein analyses we performed (**Supplementary Information X**) suggest that most group II introns are likely to encode functional reverse transcriptases. Therefore, we wondered if reverse transcriptases encoded by group II introns could be driving group I intron loss (Levra-Juillet, et al. 1989), given that the most likely mechanism of loss is reverse transcription-based retroprocessing and thus group I intron loss must rely on a heterologous reverse transcriptase(s). To test this, we looked for a correlation between group II intron presence and group I intron loss inferred from the ASRs. Specifically, we examined whether isolates inferred from the ASRs to have recent group I intron loss events have more group II introns. However, we found that isolates with recent intron losses had no more group II introns than other isolates and include several isolates with no group II introns (**Figure**). We also found that isolates lacking group II introns have significantly fewer group I introns than isolates containing group II introns (**Figure**), and that there is no significant association between group I and II intron numbers (**Figure**). This may imply that some isolates are more susceptible to intron invasion in general, but we find no evidence for a relationship between group II introns (and their reverse transcriptase activity) and intron loss.

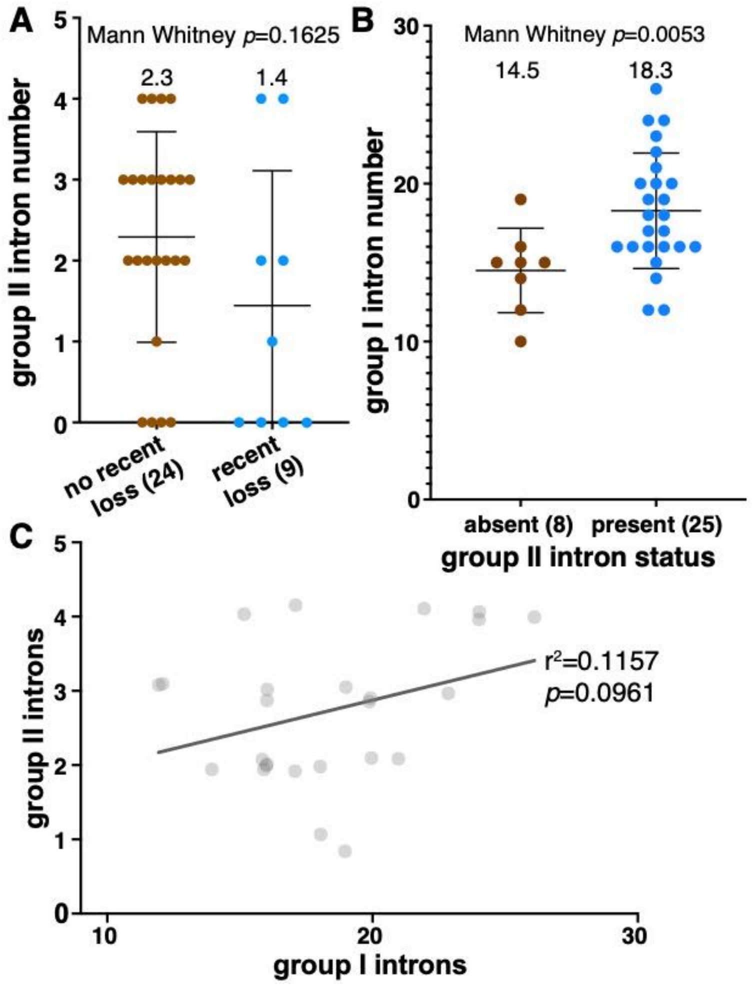

Figure. Association between group I and group II introns. (**A**) Isolates with recent intron losses (loss events involving only 1 or 2 taxa inferred from across ancestral state reconstructions using nuclear DNA, mtDNA, and presence/absence phylogenies) do not have more group II introns. The number of group II introns present in isolates with and without recent group I intron loss are plotted, and the number of isolates in each class is indicated. Means and standard deviations are indicated, with means also shown above. The recent loss isolates are *E. festucae* Fl1, *E. festucae* E1017, *E. typhina* E5073, *E. typhina* e5710, *E. clarkii* subsp. Holcus3, *E. amarillans* NFE708, *E. poae* e5101, *E. poae* e5115, *E. stromatolonga* E7543. (**B**) Fewer group I introns in isolates lacking group II introns. Group I intron numbers are plotted for isolates containing or lacking group II introns, and the number of isolates in each class is indicated. Means and standard deviations are indicated, with means also shown above. (**C**) No significant correlation between the number of group I and group II introns. Only isolates with at least one group II intron are plotted. Identical points are offset slightly for display purposes only. Correlation is linear regression.

Reference

Levra-Juillet E, Boulet A, Seraphin B, Simon M, Faye G. 1989. Mitochondrial introns aI1 and/or aI2 are needed for the *in vivo* deletion of intervening sequences. Mol. Gen. Genet. 217:168-171.
