# Supplementary material for: An Alternative Self-Splicing Intron Lifecycle Revealed by Dynamic Intron Turnover in Epichloë Endophyte Mitochondrial Genomes
Source: Mol Biol Evol. 2025 Apr 2;42(4):msaf076. doi: 10.1093/molbev/msaf076 (PMC12007492; doi:10.1093/molbev/msaf076)
Supplement: msaf076_Supplementary_Data [file Supplementary_Figure_5.pdf › Supplementary_Figure_5.pdf]

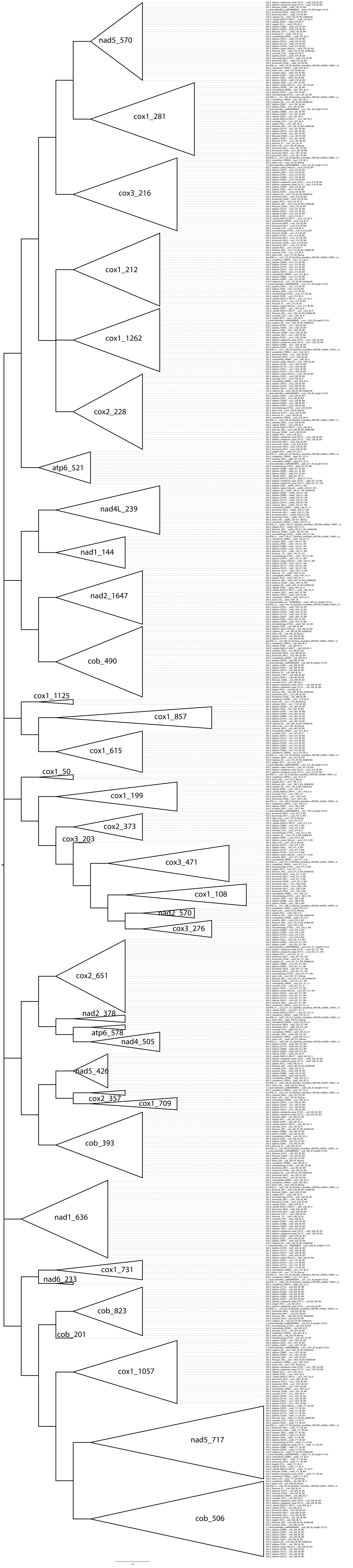

Supplementary Figure 5. Phylogeny of self-splicing introns found in *Epichloë* mitochondrial protein-coding genes. Each clade corresponds to a single self-splicing intron, the names of which are indicated, with species origin indicated to right. Phylogeny was constructed from an alignment of all intron sequences. Scale is indicated at bottom.
